# Selective (sono)photocatalytic cleavage of lignin-inspired *β*–O–4 linkages to phenolics by ultrasound derived 1-D titania nanomaterials

**DOI:** 10.1016/j.ultsonch.2024.106829

**Published:** 2024-03-02

**Authors:** Abdul Qayyum, Dimitrios A. Giannakoudakis, Dariusz Łomot, Ramón Fernando Colmenares-Quintero, Kostiantyn Nikiforow, Alec P. LaGrow, Juan Carlos Colmenares

**Affiliations:** a Institute of Physical Chemistry, Polish Academy of Sciences, Kasprzaka 44/52, 01-224 Warsaw, Poland; b Laboratory of Chemical and Environmental Technology, Division of Chemical Technology, Department of Chemistry, Aristotle University of Thessaloniki, Thessaloniki GR-541 24, Greece; c Engineering Research Institute (In^3^), Universidad Cooperativa de Colombia, Medellín 50031, Colombia; d Scientific Imaging Section, Okinawa Institute of Science and Technology Graduate University, Kunigami-gun, Okinawa 904-0412, Japan

**Keywords:** SonoPhotoCatalysis, Ultrasonication, Titania nanostructure, Biomass valorization, β-O-4 linkages

## Abstract

Catalytic conversion of lignin to value-added aromatic compounds is still an open challenge, since the selective cleavage of the linkages interconnecting the aromatic molecules, especially the β-O-4 ones, is not efficiently achieved yet. Herein, novel titania-based nanostructured materials were synthesized using low-power-low-frequency ultrasound that demonstrated high efficiency for the selective cleavage of C_α_-C_β_ bond of β-O-4 linkages of lignin-inspired model compounds. Going a step ahead, experiments of sonophotocatalytic valorization of 2-phenoxy-1-phenylethanol were contacted for the first time, where the exposure to ultrasound leading to better conversion and selectivity towards the desired products in the case of the novel ultrasound-synthesized nano-photocatalyst. Mechanistic insights showcased that photogenerated holes are the main active species in the catalytic process. In general, this research work provides a green, effective, and cost-effective approach for the selective and efficient catalytic lignin valorization.

## Highlights

• Ultrasonication was successful utilized as a synthetic tool for novel TiO_x_ photocatalyst.
• Ultrasounds led to 1-D nanostructures with interesting physicochemical features.
• Optimization of ultrasound power play a key role on the synthesis of the titania samples.
• Our novel ultrasound-derived titanate sample showed elavated β-O-4 selective cleavage.
• Sonophotocatalytic studies revealed the higher selective conversion of lignin inspired model compound to the phenolics than photocatalytic studies.

## Graphical abstract

**Figure:**
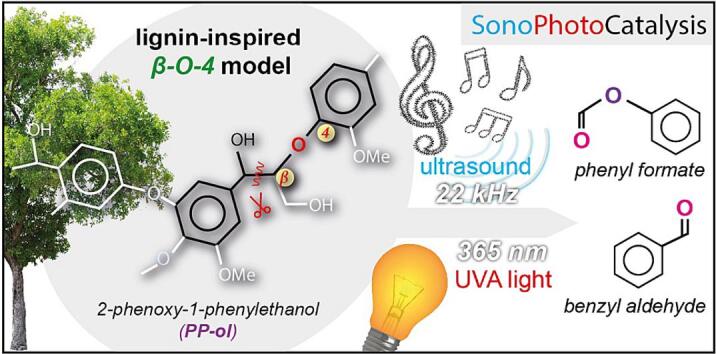

## 1 Introduction

Lignocellulose biomass is considered as the best potential abundantly available renewable feedstock for the synthesis of a wide-range of high-value products [1]. Lignocellulose is composed of cellulose, hemicellulose and lignin. Lignin is still receiving the higher level of attention comparing to cellulose and hemicellulose, due to the complexity of the structure resulted from the heterogeneteity of the linkages and the wide range of the interconnected aromatics/substituted-phenyl molecules [2], [3]. The aromatic compounds in the lignin are connected with different kind of linkages such as *β*-O-4, *α*-O-4, *β*-5, 4-O-5, 5-5′, *β*-*β* and *β*-1, with *β*-O-4 linkage to be the predominant one, (43–56 %) [4].

The controlled conversion of lignin-inspired model aromatic compounds containing *β*-O-4 linkages into mono-aromatic compounds is highly desirable for industries and bio-refineries, as lignin containing feedstocks are cheap and abundantly available in nature or/and as waste for various processes [2], [5], [6]. The selective cleavages of *β*-O-4 linkage towards obtained specific aromatic compounds is a major challenge in sustainable chemistry research [3], [7], [8]. Conventional approaches, such as acid catalysis, gasification and pyrolysis etc., for the breakage of the linkages in the lignin, typically require harsh conditions and elevate energy consumption, and in addition the cleavage to be non-selective [2], [7]. Generally, two steps approaches like peroxidation and then cleavage of *β*-O-4 linkages of lignin depolymerization may be executed under mild conditions, but this approach requires additional oxidizing/reducing agents or acid/base, which leads to the formation of a higher amount of byproducts and hazardous wastes [9], [10].

The development of a greener and more sustainable approach for the deploymerization of lignin under mild conditions is highly desirable. In general, catalysis is considered as a promising approach for the depolymerization of lignin [11]. The homogeneous catalytic break-down of lignin has been reported, although the main draw-back is that the usage of high volume of reagents/solvents that are also hazardous wastes, while in many cases high temperatures are required [12], [13]. All these issues have as an effect a high cost and complexity of processes due to the required separation and purification side-methods [14]. On the contrary, the precise design of heterogeneous catalytic possesses can overcome most of the above-mentioned drawbacks of homogeneous catalysis [15]. Most importantly and specifically, photocatalytic processes can be achieved using minimal amount of energy coming from the most abundant and renewable source of power, sunlight. Bearing in mind the uniqueness of photochemistry, our goal is to achieve elevated selective photocatalytic performance for biomass valorization to added-value chemicals without the use of additives/reagents at ambient conditions. To achieve so, the most important parameter is the design and development of novel nano-photocatalysts.

Semiconductor nanomaterials play a pivotal role for photocatalytic applications in energy storage, gas sensors, photovoltaic solar cells, hydrogen production and environmental remediation [16], [17], [18], [19], [20]. Titania based nanomaterials have been comprehensively studied for a wide range of photo-catalytic applications after the reporting of Fujishima and Honda’s pioneering work on photoelectrochemical water splitting using TiO_2_ [21]. Titania-based nanostructures are being widely and intensively studied for the photo-catalytic degradation of various pollutant compounds in aqueous matrixes. However, unselective conversion of the organic molecules to the corresponding products limited their use in the field of organic synthesis, which the key required aspect is selectivity of photoreactivity in order to eliminate the formation of any possible by products and side reactions and so to achieve high yield of the desired compound. By modifying the physicochemical properties, and more importantly the textural, optical and surface chemistry characteristics by tuning and optimizing the synthetic protocol, the reactivity of the titania nanostructures can be manipulated on demand, which is still a challenging research task but is highly interesting [22].

The utilization of ultrasound (US) as a tool and source of mechanochemical power in nanomaterial synthesis has been presented as a prosperous strategy towards novel nano-catalysts [23]. In general, the irradiation of US waves in the liquid phase creates unique low and high-pressure zone due to the cavitation formation and collapse. The collapsing of these cavitations’ leads to the formation of the hotspot, where the pressure and the temperature can reach up to 1000 bars and 5000 K, respectively [22], [24]. The utilization of US also results in physical effects like elevated micro-mixing, mass transfer, and fragmentation/deaggregation of particles predominately due to the formation of microjets [25].

In this research work, a green and sustainable approach was utilized during the synthesis of novel titania-based nanostructure via the precipitated method, utilizing ultrasound irradiation (US) as a synthesis tool. The key objective was to investigate if and how the power of the utilized low frequency (22 kHz) US can effect on demand the physicochemical properties towards elevated photocatalytic activity. In order to have a better insight regarding the effect of US on the physicochemical features, materials were synthesized using 5 different US powers and in silent conditions (without US irradiation and using magnetic stirring). For the sake of comprehensive comparisons, the benchmark commercially available TiO_2_ nanoparticles (P25) were also studied. The key goal was to determine if the synthesized nanomaterials possess a higher catalytic activity towards the cleavage of C-C bond of a lignin-inspired bi-aromatic model molecule, 2-phenoxy-1-phenylethanol (PP-ol), with higher yield of the desired mono-aromatic compounds and their oxidation counterparts. The potential pathways and involved mechanisms for this catalytic cleavage of C-C bond of PP-ol and the formation of the corresponding products was also explored. Going a step even further, we studied, for the first time to the best of our knowledge, if ultrasound irradiation can be utilized as a catalytic process intensification tool in order to enhance the photocatalytic performance and to avoid the use of mechanical magnetic stirring.

## 2 Experimental part

### 2.1 Materials

The chemicals and materials used were titanium isopropoxide (TTIP, 98 %, Acros Organics), isopropanol (99,7 %, POCH), sodium hydroxide (NaOH, ChemPure), benzyl alcohol (99.5 %, ChemPure), benzyl aldehyde (99 %, ChemPure), acetonitrile (AcN, HPLC grade, POCH), 2-Phenoxy-1-phenylethanol (95 %, abcr), 2-Phenoxy-1-phenylethanone (97 %, Sigma Aldrich), phenyl formate (≥98, Sigma Aldrich), phenol (99 %, Alfa aesar), potassium iodide (Chempur) oxalic acid (OA**)** (98 %, Alfa Aesar), 1,4-Benzoquinone (≥98, Sigma Aldrich), and silver nitrate (Ag) (99.8 %, Stanlab), and TiO_2_ (P25, Evonik Degussa). All the chemicals were used as received. Mili-Q water was used conclusively.

### 2.2 Synthesis of the catalysts

**Scheme 1:**
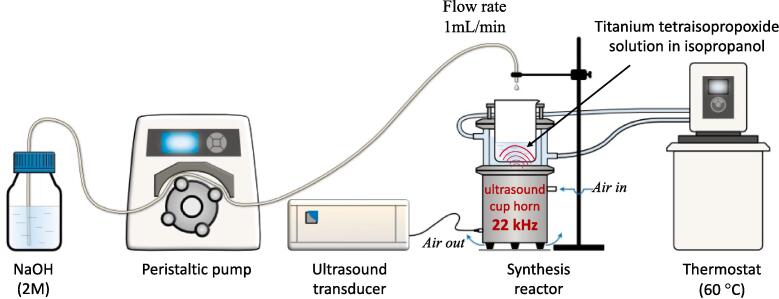
Schematic illustration of the synthesis setup and details for the key parameters.

The synthesis of titania based nanomaterials was performed by taking the 20 mL solution of titanium isopropoxide in isopropanol with the ratio of 1:3 in a reactor (glass beaker, 250 mL). This solution was kept inside the cuphorn sonicator of 22 kHz (Sinaptec, NexTgen Lab500 generator). 100 mL of sodium hydroxide (2 M) solution (in water) was added in the above solution with a control flow rate of 1 mL/min. The temperature was stabilized at 60 °C during the ultrasound irradiation. After the complete addition of 100 mL of sodium hydroxide solution, a white dispersion was obtained, which was further filter by pre-acidified Whatman paper (Rotulabo®-Roundfilter) followed by dilute HCl and mili Q water till obtaining the neutral pH of the filtrate. A solid residue was obtained after drying the filtrate at 90 °C for 16 h in an oven. The synthesis procedure is shown in Scheme 1.

For this synthesis, a series of nanomaterials were synthesized by altering the power (8, 24, 40, 56 and 72 W) of 22 kHz sonicator. The synthesized nanomaterials, referred to as US22-representation depending on the utilized power, are summarized in Table 1. A sample was synthesized by the same above mention protocol by using magnetic stirring (200 rpm) instead of ultrasound in order to study the effect of ultrasonication on the material synthesis.

**Table 1:** The followed ultrasound parameters and the abbreviations of the synthesized materials.

Serial No.	Ultrasound (22 kHz) power (W)	Samples name
1	8	US22-A
2	24	US22-B
3	40	US22-C
4	56	US22-D
5	72	US22-E
6	Magnetic stirring (200 rpm)	MagS

### 2.3 Physicochemical characterizations

The textural properties of the synthesized nanomaterials were evaluated based on nitrogen physisorption/desorption experiments. These measurements were performed by using Micromeritics instrument (ASAP 2020) at −196 °C. The degassing of the samples was performed at 90 °C under the vacuum for 6 h before the measurement. The surface area was estimated by using Brunauer–Emmett–Teller (BET) method. The pore size and volume were calculated by using the Barret-Joyner-Halenda (BJH) method.

The defuse reflectance spectra (DRS) of all these synthesized samples was studied in the range of 250–900 nm by using the instrument a Jasco V-570 spectrophotometer. The base line was obtained by using [poly(tetra- fluoroethylene)].

The crystallinity of all these synthesized samples was investigated by the powder X-ray diffraction method by using Siemens D5000 diffractometer (40 kV and 40 mA), which was equipped with a horizontal goniometer. The morphology of the synthesized samples was studied by using Transmission electron microscopic (JEOL JEM 2100), which was operated with a LaB6 filament at 200 kV. The sample for this measurement was prepared by sonicating the samples in the ethanol solvent followed by depositing them onto 200 mesh carbon coated grid (Ted Pellam Inc.). The weight loss of all the herein studied samples was investigated by the thermal gravimetric analysis (MettlerToledo TGA/DSC^3+^) by using a thermos-balanace. The surface pH of the samples was estimated by dispersing 100 mg of the each sample in 50 mL of mili Q water (in a separate vessels sealed with parafilm), mixing in the dark for 16 h for equilibration, and then measuring the pH.

The surface compositions of all the samples was investigated by the X-ray photoelectron spectroscopy (XPS) (PHl 5000 VersaProbe™ Scanning ESCA Microprobe). X-ray source (Al-*K*_*α*_ radiation; hν = 1486.6 eV) was operated at 15 kV, 25 W and 100 µm spot size to obtain the CPS spectra. High-resolution XPS spectra were obtained by an analyzer pass energy of 23.5 eV and an energy step size of 0.1 eV. Multipak PHI software was used to analyzed the survey spectra while Casa XPS software (v.2.3, Casa Software Ltd, Wilmslow, United Kingdom) was used for the deconvolution and quantification of obtained spectra.

Temperature-programmed desorption (TPD) and temperature-programmed oxidation (TPO) measurements was performed in tubular quartz flow reactor and the concentration of evolved gas was analyzed by using mass spectrometer Dycor Dymaxion with range 1–200 *m*/*z*. Prior to TPD measurement, the samples (∼20 mg) were calcined in helium atmosphere at 100 °C. During the TPD measurement, the samples were heated at rate 10 °C/min under helium flow with the flow rate of 25 mL/min controlled by MFC GFC (Aalborg) till 700 °C. After TPD measurement, the sample was cooled down to the room temperature, and then this sample was used for the TPO measurement. TPO measurement was performed by heating at rate 10 °C/min the samples up to 700 °C in mixture of helium (99.999 %), and the synthetic air for FID 2 % O_2_/(He + N_2_) with the flow rate of 25 mL/min controlled by MFCs GFC (Aalborg).

### 2.4 Photocatalytic activity evaluation tests

The additive free photocatalytic performance of the synthesized titania samples and also commercially available TiO_2_ samples P25 Evonik was studied for the partial selective oxidation of benzyl alcohol (BnOH) or 2-Phenoxy-1-phenylethanol (PP-ol) under ultraviolent (UV, 365 nm) light irradiation produced by light emitting diodes (LEDs) (LEDMod, Omicron, Germany. 15 mg of each sample was suspended in a reactor containing the targeted substrate in the acetonitrile solvent at a stabilized temperature 30 °C using water-bath and circulating water (ultrabath Julabo DB-5(Corio CD-B5)). The suspension was kept homogenous by magnetic stirring (600 rpm), and the reactor was covered with aluminum foil to avoid any external light irradiation. The suspension was stirred under dark conditions for 1 h in order to stabilization/equilibration and to determine the adsorptive capability of the materials. After 1 h of stabilization, the lamp (100 W/m^2^ measured by radiometer (Delta OHM, HP2302.0)) was switched on. The samples for the analysis were collected prior to light irradiation and after the specific interval of light irradiation, followed by immediately filtered by a syringe filter of (0.20 µm) pore size. While the sonophotocatalytic experiments were performed by coupling the US (22 kHz with 30 µm amplitude) to the photocatalytic setup by replacing the magnetic stirring (Scheme S1).

Selected samples were calcined at 500 °C (ramp of 10 °C/min) using a muffle furnace in order to explore possible alterations on the photocatalytic performance. The reusability photocatalytic activity was evaluated using the separated powder after decantation, washing with water and decantate for multiple times and drying at the end at 90 °C for 16 h. The same procedure was adopted for up to 5 subsequent runs. In order to study the potential pathway of the conversion of PP-ol to the corresponding products, the photocatalytic activity of the herein samples was studied by using various substrates such as 2-Phenoxy-1-phenylethanone (PP-one), phenyl formate (Ph-OCHO), phenol (Ph-ol). A series of photocatalytic experiments was also performed by the addition of scavenger for various active species in order to identify the major active species that can play a key role for the photocatalytic selective cleavage of C-C bond of the PP-ol. The concentration of the scavenger substance per initial concentration (1.5 mM) of PP-ol substrate was kept equimolar (1:1). The utilized scavenger compounds such as potassium iodide (KI and) oxalic acid (OA) as hole (h^+^) scavenger, 1,4-Benzoquinone (BQ) as (O_2_^•-^) scavenger, *tert*-butanol (t-BtOH) as hydroxyl (HO^•^) and silver nitrate (Ag) as electron (e^-^) scavenger.

The obtained samples during the photocatalytic experiments were analyzed by Gas Chromatograph (GC) (Shimadzu GC-2010), which is equipped with a flame ionization detector. The capillary column (Zebron ZB-5MS, Phenomenex USA) containing a length of 30 m, diameter of 0.25 mm and film thickness of 0.5 μm was used, whereas helium was used as the carrier gas. 1 μL of the collected sample was injected with a split ratio of 8. The temperature of the column was initially maintained at 50 °C for 3 min, and later, it was increased to 300 °C with the increasing rate of 9 °C/min and hole the final temperature for 2 min.

The parameters such as conversion of substrate, yield of products and aromatic balance photocatalytic activity was calculated. These parameter for the photocatalytic partial selective oxidation of monoaromatic alcohol (BnOH) was calculated based on the following equation:

$$Conversion\left(\%\right)=\left[\frac{\mathrm{Co}-Cr}{\mathrm{Co}}\right]*100$$

$$Yield\left(\%\right)=\left[\frac{\mathrm{Cp}}{\mathrm{Co}}\right]*100$$

$$Selectivity\left(\%\right)=\left[\frac{\mathrm{Cp}}{\mathrm{Co}-Cr}\right]*100$$

$$\mathrm{Aromatic}\;Balance\left(\%\right)=\left[\frac{\mathrm{Cr}+Cp}{\mathrm{Co}}\right]*100$$

where C_o_ is the initial concentration (mM) of BnOH, while Cr is the concentration of BnOH, whereas Cp is the concentration of the Ph-CHO, respectively, at a selected specific time interval of each photocatalytic reaction.

For analysis of collected samples from of the bi-aromatic substrate such as PP-ol, the initial temperature of the GC column was set at 40 °C for 3 min and increased to 280 °C at a rate of 10 °C/min without holding time, and its further increase to 300 °C at a rate of 20 °C/min with the final holding time of 5 min.

The results of conversion of PP-ol, the yield of products such as benzyl aldehyde (Ph-CHO), phenyl formate (Ph-OCHO), phenol (Ph-ol) and 2-Phenoxy-1-phenylethanone (PP-one), were calculated based on the following equations [26].

$$\mathrm{ConversionofPP}-ol\left(\text{\textbackslash{}\%}\right)=\left[\frac{\text{Moles of reacted substrate (}PP-ol)}{\text{Moles of initial added substrate (}PP-ol)\;}\right]*100$$

$$YieldofPhCHO\left(\text{\textbackslash{}\%}\right)=\left[\frac{\mathrm{MolesofformedPhCHO}}{\text{Moles of initial added substrate(}PP-ol)\;}\right]*100$$

$$YieldofPhenylformate\left(\text{\textbackslash{}\%}\right)=\left[\frac{\mathrm{Molesofformedphenylformate}}{\text{Moles of initial added substrate(}PP-ol)\;}\right]*100$$

$$YieldofPhol\left(\text{\textbackslash{}\%}\right)=\left[\frac{\mathrm{MolesofformedPhol}}{\text{Moles of initial added substrate(}PP-ol)\;}\right]*100$$

$$\mathrm{YieldofPP}-one\left(\text{\textbackslash{}\%}\right)=\left[\frac{\mathrm{MolesofformedPP}-one}{\text{Moles of initial added substrate(}PP-ol)\;}\right]*100$$

$$\text{Aromatic}\;\text{balance}\left(\%\right)=\left[\frac{\begin{array}{c} 2\left(\;\text{moles of reacted PP - ol}\right)+\text{moles}\;\text{of}\;\text{PhCHO}\\ +\text{moles of Phol}+2\left(\text{moles of pp - one}\right) \end{array}}{2\text{\textbackslash{}\{ Moles of initial added substrate(PP - ol)\textbackslash{}\}}\;}\right]*100$$

## 3 Results and discussion

### 3.1 Characterizations

**Fig. 1:**
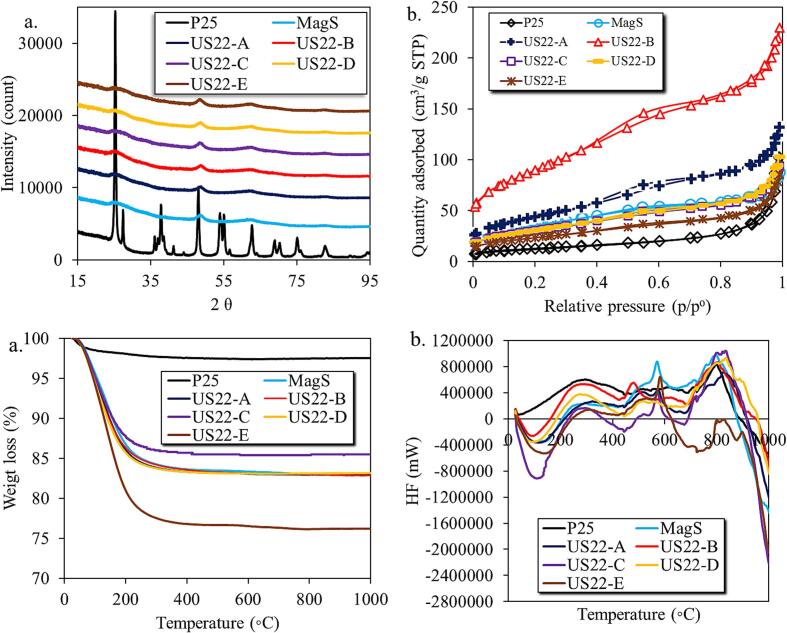
The XRD patterns (a), N_2_ sorption isotherms (b), thermogravimetric analysis profiles (c), and differential thermal analysis (d) for P25, MagS and US-assisted synthesized titania nanomaterials by 22 kHz with different powers.

The crystallographic nature of all the synthesized and commercial samples was studied by the X-ray diffraction (XRD) method, with all the obtained patterns to be collected in Figure 1a**,** while the calculated crystallite sizes, based on Scherer method [27], to be presented in Table S1. The P25 sample showed the presence of anatase and rutile phases which are found to be similar as reported previously [28], [29]. The patterns of all the synthesized samples did not reveal well-defined reflections, a fact linked either to an amorphous nature or/and to very nanoscaled size of crystals. The broad peaks of a very low intensity, appeared with maxima at 25, 45 and 63° 2θ, are assigned to anatase phases [22], [30].

The textural properties of all samples were studied by N_2_ sorption experiments, with the isotherms to be collected in Figure 1b and the derived parameters in Table 2. The first outcome is that the use of low-power ultrasound irradiation during the synthesis resulted in higher porosity as compared to higher-power of ultrasound. The highest specific surface area (S_BET_) was found for US22-B, reaching 319 m^2^/g. This value can be assumed as very high for metal oxides, and it is almost 7 times higher than that of P25 and 2.5 times higher than the sample obtained in silent conditions (MagS). The values of total pore volume (V_Tot_) showed a similar trend as the S_BET_ did, with US22-B presented the highest V_Tot_ (0.339 cm^3^/g) followed by US22-A sample (0.193 cm^3^/g). The variation of the S_BET_ for the samples prepared under US irradiation between 82 to 319 m^2^/g suggest that the US irradiation affect directly the crystallization process, both physically and chemically.

**Table 2:** Textural and optical parameters of all the herein studied materials.

Index numb.	Material	Specific surface area (m^2^/g)	Pore volume V_p_ (cm^3^/g)	Pore size d_p_ BJH (nm)	Estimated band gap (eV)	Surface pH
1	P25 (Evonik)	47	0.139	13.8	3.22	6.2
2	MagS	125	0.128	4.5	3.44	8.0
3	US22-A	156	0.193	5.0	3.46	8.0
4	US22-B	319	0.339	4.5	3.37	7.4
5	US22-C	109	0.136	5.2	3.48	8.5
6	US22-D	109	0.146	5.5	3.47	8.4
7	US22-E	82	0.120	6.3	3.45	8.6

The outcomes of XRD patterns as well as our previous findings suggested that utilization of low power US lead to higher in values textural features (porosity). This can be ascribed to enhanced chemical heterogeneity, and especially to elevated amount of surface hydroxyl groups of synthesized samples [24], [31] as it will be discussed herein after. The obtained N_2_ adsorption/desorption isotherms (Figure 1b) revealed to be complex for US22-A and US22-B, suggesting the presence of pores with a wide range of sizes. More specifically, micro-, meso-, and macro-pores exist. In the case of US22-C, US22-D, US22-E, MagS and P25 samples, the isotherms were revealed to match better the Type I(b) and Type II [31], [32], suggesting that these samples possess mesopores predominately, with diameter in the range of 2–9.5 nm, and micropores with diameter in the range of 1.6 to 2 nm, as can be seen and at the pores size distribution plots (Figure S1**)**.

The weight loss profiles and the thermal stability of all the samples were examined by differential thermal gravimetric analysis. P25 showed a very low weight loss (≤2.5 % till the temperature 400 °C) as displayed by Figure 1c**,** whereas all the synthesized samples showed more than 15 % weight loss. The highest weight loss was revealed by the US22-E sample. This higher weight loss in the case of synthesized samples suggested the presence of a higher amount of water or/and higher density of surface hydroxyl groups. The higher porosity and possible hydrophilicity due to the surface OH groups compare to P25 can also be a detrimental reason for the elevated water adsorption [33]. It should be pointed out that no calcination at high temperature was performed in order to keep the synthetic protocol as green as possible. In addition, the targeted application of the herein presented samples is close to room temperature, while the presence of pre-adsorbed humidity can be beneficial for the formation of active reactive oxygen species (ROSs).

Thermal treatment above 400 °C revealed some possible phase transformations for all samples but not for US22-E. From the differential thermal analysis (DTA) profiles in Figure 1d, it can be seen an intense endothermic process up to 200 °C for the synthesized samples, suggesting the removal/desorption of water, as suggested by DTA plots [6]. Whereas this shape trend didn’t observe in the case of P25 sample, which supports the former suggestion in the case of synthesized samples. Another weight loss for all samples was observed from 200 °C to 450 °C, corresponding to an exothermic process. This exothermic process might suggest the removal of other possible carbon containing functional groups [34]*.* The peaks in the temperature range from 450 °C to 600 °C can be assigned to phase transformation from amorphous to rutile phase [10], whereas the transformation of anatase to rutile phase was observed in the temperature range from 600 °C to 900 °C [35].

Temperature program desorption (TPD) measurement of the MagS, US22-B and P25 samples was studied which showed that the synthesized MagS and US22-B samples contain water on the surface in a higher amount compared to P25 (Figure S2). These results are in a good agreement with the thermal analysis results. Temperature program oxidation (TPO) of the samples after TPD was also performed. The results (Figure S3) showed that MagS and US22-B showed evolution of CO_2_, while in case of P25 did not showed elution of CO_2_. This evolution of CO_2_ from the synthesized samples suggested that these samples contained the carbonaceous species on the surface.

Fourier transform infrared (FTIR) measurements of all the herein samples were performed in order to identify the bonds of specific functional groups present or not on the samples’ surface, with the obtained results to be presented in the Figure S4. A broad band below ∼ 1200 cm^−1^ appeared for all herein samples, which corresponding to the characteristic vibrational mode of TiO_2_ (Ti–O stretching mode of Ti–O–Ti) [36]. MagS and US22-B samples showed a broad band with maximum around ∼ 3221–3340 cm^−1^ corresponding to the –OH group, due to the presence of water and in good agreement with TPD and thermal analysis. For MagS and US22-B samples, the absorption band appeared at around 1615–1635 cm^−1^ may correspond to two different types of groups. It may corresponded either to –OH bending modes of adsorbed water on the surface of TiO_2_ [37], [38], or to the antisymmetric stretching vibrations of carboxylate containing groups (range of 1650–1510 cm^−1^) [39].

The optical properties of all the herein studies samples were evaluated based on ultraviolet–visible diffuse reflectance techniques in wavelength range of 250–900 nm. All the synthesized samples showed light absorption in the ultraviolet region below 370 nm, whereas P25 sample revealed light absorbance below 430 nm (Figure S5a). These results indicated that photo-catalytic activity of all these synthesized samples could be possible only under ultraviolet light irradiation. The bandgaps (*E_g_*) were estimated by the Kubelka–Munk approach [40]. As it can be seen in Figure S5b**,** all the synthesized samples possessed a slightly higher bandgap than that of P25 sample (3.2 eV). The lowest band gap (3.37 eV) among the synthesized samples was found for US22-B while the highest (3.47 and 3.48 eV) for US22-D and US22-C samples, respectively.

**Fig. 2:**
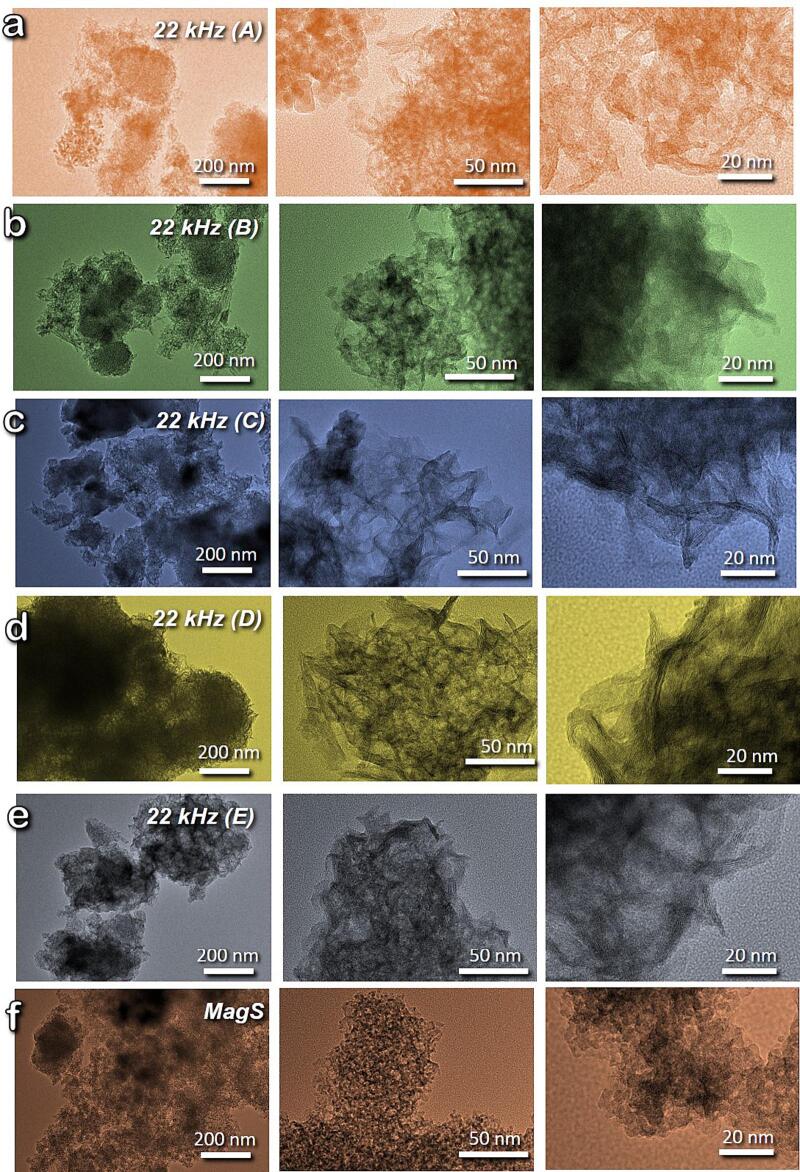
TEM images of the synthesized titania samples.

The morphological features of all the synthesized samples were explored by transmission electron microscopy (TEM) with the obtained images collected in Figure 2. The first outcome is that exposure to ultrasound waves during the synthesis leads to the formation of different morphologies compared to silent condition, resulting predominately to 1-dimensional nanorod-like shaped nanostructures. Another outcome is that increasing the power of the utilized frequency led to increment of the nanorods' length. The US22-A sample, which was synthesized using the lowest power (8 W) of 22 kHz showed the highest level of amorphicity in shape, with the detected nanorods to 15–40 nm in length, whereas the US22-E sample, which was synthesized using the highest power (72 W) showed the formation of nanorods of 30–70 nm length. All the ultrasonic-assisted synthesized samples showed the formation of cobweb-like structure by the aggregation of nanorods. Another, interlayer spacing was also observed between the nanorods, which might be indicative of titanite nanosheets [41]. Whereas in the case of MagS sample, no formation of 1-dimensional nanorods was observed. MagS sample showed the formation of nanoparticles of the spherical shape of size 3–8 nm.

The surface chemistry of all the synthesized and commercial P25 samples was analyzed by the X-ray photoelectron spectroscopy (XPS). The high-resolution deconvoluted spectra and the obtained atomic percentages for O *1 s*, C *1 s*, Ti *2p* and Na *2p* are presented in the Figure S6-S7 and Table 3, respectively. Deconvolution of the high-resolution core spectra of O *1 s* region for all the herein studies samples showed two peaks in the range of 530.0–532.0 eV and 531.0–532.0 eV. The former one represents the bridging oxygen (Ti-O-Ti) of the metallic oxide, while the lateral one is linked to the surface hydroxyl (−OH) group or/and C-O bonds [42], [43]. Deconvolution of the high-resolution spectra of Ti *2p* region for all samples discover single chemical state of titanium with doublet peaks corresponding to Ti *2p 3/2* (459.2 eV) and Ti *2p ½* (464.9 eV) [42], [44]. Deconvolution of high-resolution spectra of C *1 s* core energy of all the samples showed three peaks in the ranges of 284.6–284.9 eV, 286.0–286.8 eV and 289.5–259.9 eV which correspond to the presence of adventitious carbon, epoxide C-O-C and carboxyl (O-C = O) functional groups [43], [45]. All the synthesized samples revealed the presence of sodium which might be due to the using high concentrated sodium hydroxide during the precipitation method. Whereas in the case of P25 sample, sodium was not detected.

**Table 3:** The atomic percentages of O *1 s*, C *1 s*, Ti *2p* and Na *2p* for synthesized and commercial P25 samples.

	O*1s*	O*1s*	Ti *2p 3/2*	Ti 2p *1/2*	C *1 s*	C*1s*	C *1 s*	Na *2p*
	(Ti-O-Ti)	(Ti-OH /C-O)			(C-C)	(C-O-C)	(COOH/ COOR)	
	530.7	532.0	459.2	464.9	284.8	286.2	289.6	31.5
US22-A	54.8	7.5	16.7	8.4	2.3	0.7	0.7	8.9
US22-B	59.1	6.0	17.8	9.0	2.3	0.8	0.7	4.3
US22-C	55.6	4.2	16.2	8.1	3.3	0.5	0.7	11.4
US22-D	57.7	4.4	16.6	8.3	2.0	0.3	0.5	10.2
US22-E	56.5	4.2	16.0	8.0	2.5	0.4	0.7	11.7
MagS	57.7	4.1	16.3	8.1	2.6	0.5	0.9	9.8
TiO_2_ P25	58.8	4.4	17.2	8.6	8.7	1.1	1.2	---

### 3.2 Photocatalytic partial selective oxidation of benzyl alcohol to benzyl aldehyde

The photocatalytic performance of the synthesized samples was initially evaluated by the partial selective oxidation of BnOH to Ph-CHO at ambient conditions without the addition of any oxidative reagent. The commercially available P25 is a well-known benchmark material with an elevated photocatalytic degradation ability of a wide range of organic emergent pollutants, with its reactivity to be harsh and unselective transformation of the organic pollutants, mostly to mineralization [26], [42]. For organic compound synthetic processes and/or selective redox conversion to specific compounds, the selectivity and yield are the key parameters. As we previously reported, P25 possesses a great photocatalytic conversion of BnOH rate and extend, but its selectivity towards the desired Ph-CHO is low, since the selectivity and yield to be only 36 % and 35 %, respectively, after 6 h of light irradiation [31]. So, our goal was to synthesized novel titania photocatalyst of a higher selectivity and yield toward Ph-CHO upon the partially oxidation of BnOH.

**Fig. 3:**
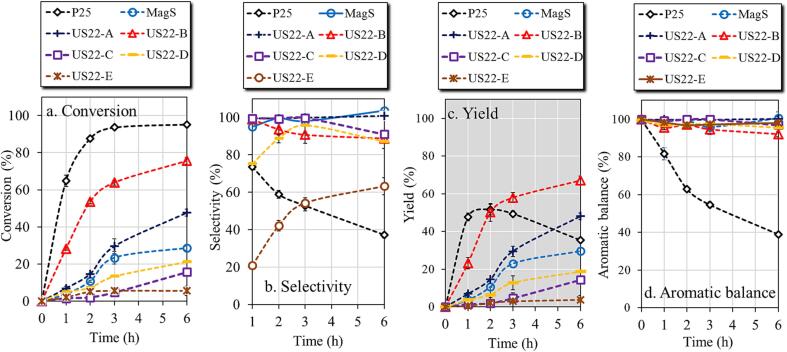
Photocatalytic activity of P25, MagS and synthesized titania nanomaterials by ultrasonic assistance of 22 kHz with different power for the selective oxidation of benzyl alcohol to benzyl aldehyde.

The 22 kHz ultrasonic-assisted synthesized titania by using different power showed a wide variation of photocatalytic performance **(**Figure 3**)**. The first outcome was that samples synthesized by using low power US showed better photocatalytic performance as compared to samples synthesized by using high power US. More specifically, US22-B showed the highest and fastest conversion of BnOH to Ph-CHO, with the BnOH conversion reaching 75 % and the Ph-CHO yield 67 %, after 6 h of light irradiation. US22-A also showed a good BnOH conversion (46 %) and the selectivity toward Ph-CHO was found to be more than 98 %. The third photocatalytic performance was presented by US22-D, which showed 20 % BnOH conversion and 20 % Ph-CHO yield. Higher power of US lead to materials of almost negligible photocatalytic performance.

The US-assisted synthesized nanomaterials using different powers showed variations on the photocatalytic partial selective oxidation of BnOH to Ph-CHO performance, which can be linked to the different textural and morphological features, as well to the different chemical surface compositions. The use of low power, such as 8 and 24 W of the 22 kHz resulted in the materials with the highest porosity, with the specific surface area reaching up to 319 m^2^/g. The further increase of the power of utilized US during the synthesis led to a lowering in the porosity of the synthesized materials. Also, the elevated pore size of the low power of US-assisted synthesized samples was observed to be higher than the high power of US, which can have a positive effect on the penetration of BnOH molecules via the pores towards the active catalytic sites. In addition, the increased porosity leads affect positively the adsorption of the BnOH molecule on the surface of titania nanomaterials, resulting in elevated photocatalytic activity [31]. Furthermore, the formation of 1-dimensional structures of the US-assisted synthesized samples helps to improve the harvesting of the light and hence the photocatalytic conversion of BnOH. In the case of low-power US-assisted synthesized samples, the formation of cobweb-like structure was also observed, which can also lead to the diffusion of the BnOH molecules to the interior portion of the nanomaterials, which can also elevate the BnOH conversion as the contact area for the catalytic reaction is increased [24].

Except the textural and morphological features, two other features can be the reasons behind the highest photocatalytic efficiency of US22-B. The first is the highest amount of pre-adsorbed water. These water moieties can be transformed to reactive oxygen species (ROSs) promoting redox reactions and the cleavage of the carbon bond. The second aspect that should be considered is the increased surface chemical heterogeneity, since US22-B presented the highest amount of oxygen and carbon containing surface functional groups as well as the lowest amount of Na [23], [24].

### 3.3 Photocatalytic oxidation of 2-Phenoxy-1-phenylethanol

Among the 22 kHz ultrasound-assisted synthesized materials, US22-B showed the highest conversion of BnOH and the highest yield of Ph-CHO and hence, it was selected among the rest of samples for the catalytic selective upgrade of the lignin-inspired model compound 2-phenoxy-1-phenylethanol (PP-ol) by the selective cleavage of the C_α_ − O, C_α_ − C_β_, or/and C_β_ − O bonds of the *β*-O-4 linkages. For the sake of optimizations (PP-ol concentrations and catalyst loading) and comparisons with US22-B, P25 and MagS were also tested.

The results of the experiments using different PP-ol initial concentrations (ranging from 0.5 mM to 2.5 mM) by using 1 g/L of catalyst are collected in the Table 4. The analysis by GC after the exposure to UV light for 6 h revealed the formation of four products, namely PP-one, Ph-CHO, Ph-ol and Ph-OCHO. The formations predominately of these products are also reported in the literature by using different catalysts [3], [43]. The first outcome about the initial concentration optimizations was that the PP-ol conversion declines by the increment of PP-ol initial concentration. Since Ph-CHO and Ph-OCHO are the majorly desired product from PP-ol deploymerization due to their potential usage for various industrial applications/synthesis, the focus regarding the photoreactivity evaluation was based on the yield of these compounds. Hence, the 1.5 mM initial PP-ol concentration was found as the optimum one, with the yield of Ph-CHO to be 25 % and of Ph-OCHO 29 % with an acceptable PP-ol conversion of 69 %. The aromatic balance was also comparatively elevated (74 %). It should be mentioned that the light irradiation with absence of catalysis (photolysis test) did not reveal the decomposition of PP-ol, suggesting its stability upon UV exposure.

**Table 4:** Optimization of different concentrations of PP-ol for the photocatalytic activity of P25 (1 g/L).

Concentration of PP-ol (mM)	Conversion of PP-ol (%)	Yield (%)				Aromatic balance
		Ph-CHO	Ph-OCHO	Ph-ol	PP-one	
0.25	83	12	11	4	17	47
0.5	83	20	14	16	11	54
1	76	23	21	23	8.3	64
1.5	69	25	29	20	6.2	74
2	59	23	14	15	4	77
2.5	60	24	15	15	4.5	78

Catalyst loading 1 g/L, magnetic stirring 600 rpm, 6 h of UV light irradiation

To study the effect of different catalyst loadings, a series of experiments were performed by varying the catalyst amount from 0.5 to 4 g/L by using the optimized PP-ol initial concentration (1.5 mM) and the results are shown in Table 5. A general observations from these results is that increasing of the photocatalyst loading above 1 g/L has a negligible impact on the PP-ol conversion but a negative one on the Ph-OCHO yield and total aromatic balance. The highest yields of Ph-CHO (25 %) and Ph-OCHO (29 %) were found using 1 g/L catalyst loading and so this loading was determined and used herein afterwards as the optimum one.

**Table 5:** Optimization of different catalytic loading of P25 for photocatalytic conversion of PP-ol to the corresponding products.

Catalyst loading (g/L)	Conversion of PP-ol (%)	Yield (%)				Aromatic balance
		Ph-CHO	Ph-OCHO	Ph-ol	PP-one	
0.5	59	20	28	11	5.6	76
1	69	25	29	20	6.2	74
2	70	24	21	19	5.4	67
3	74	22	23	22	7.2	70
4	75	26	16	23	7.2	65
5	60	26	2	13	6.02	60

Concentration of PP-ol 1.5 mM, magnetic stirring 600 rpm, 6 h of UV light irradiation.

**Fig. 4:**
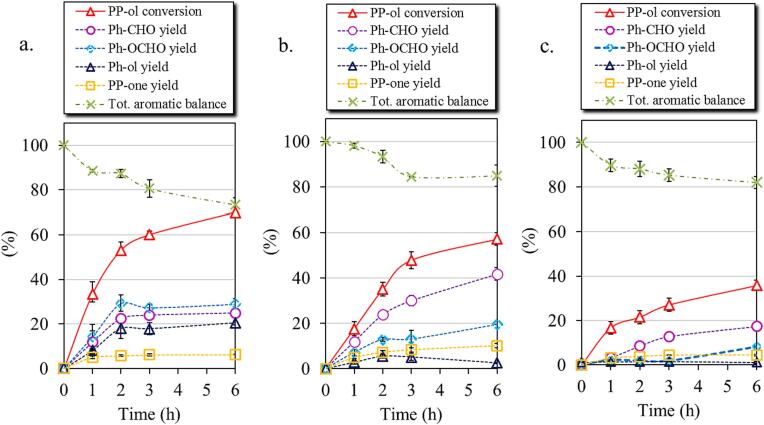
Photocatalytic activity for the oxidation of 2-phenoxy-1-phenylethanol to the corresponding products by using commercial P25 (a), US22-B (b), and MagS (c) samples (1.5 mM PP-ol in acetonitrile, catalyst loading 1 g/L, magnetic stirring 600 rpm, 6 h of UV light irradiation).

The evolution of the photocatalytic results for US22-B, MagS, and P25 using the optimum parameters (1.5 mM and 1 g/L) are presented in Figure 4. The commercial P25 showed the highest PP-ol conversion (69 %) after 6 h of exposure to low-power UV light, with the yields of Ph-CHO (25 %), Ph-OCHO (29 %), Ph-ol (20 %) and PP-one (6 %) to be low. The aromatic balance was found the smallest one (74 %), suggesting a low selectivity. This can be link also to the fact that P25 revealed the ability to decompose benzyl alcohol and benzaldehyde, as presented above and in previous works [22], [24]. Whereas US22-B sample showed a slightly lower conversion of PP-ol (57 %) than P25, the yield of the targeted products were significantly higher, 42 % for PhCHO and 20 % for Ph-OCHO. The yields of Ph-ol and PP-one were found 2 and 10 %, respectively and the total aromatic balance 84 %, which is higher by ± 14 % than that of P25. The MagS sample showed dramatically low PP-ol conversion (36 %) and yields towards Ph-CHO (17 %), Ph-OCHO (8 %), Ph-ol (2 %), and PP-one (4.4 %). Considering all the above, it is apparent that the material obtained utilizing the 22 kHz ultrasonic power after optimization outperformed the rest significantly.

Even though the existing works in the literature regarding the photocatalytic valorization of PP-ol are limited, the results for other materials studied for PP-ol conversion are collected in Table 6. It can be seen that the ultrasound-assisted herein synthesized nanomaterials outperform all the reported materials so far.

**Table 6:** Conversion of PP-ol to corresponding products using various catalysts.

Catalyst	PP-ol	PP-one	Ph-CHO	Ph-OCHO	Ph-OH	Ph-COCH_3_	Experimental conditions	References
TiO_2_ (homemade)	12	1	–	–	–	–	10 mg of catalyst, 1 mL of CH_3_CN, 455 nm, 10 h	[43]
Pd/TiO_2_	19	–	–	–	–	–	0.1 mmol of PP-ol, 5 mg of catalyst, 1.0 mL of CH_3_CN, 455 nm, 42 °C, 4 h. The	[26]
VOSO_4_	44.6	44.6	–	–	–	–	Substrate (0.5 mmol), catalyst (0.1 mmol, MeOH (2 mL), O_2_(0.4 MPa), 100 °C, 12 h	[44]
Zn_4_In_2_S_7_-GO	38.5	34.3	–		2.3	1.85	0.1 mmol of PP-ol, 0.01 g of catalyst, O_2_, 5 mL of CH_3_CN, 300 W Xe-lamp, for 2 h.	[45]
TiO_2_ P25	69	6.2	25	29	20	–	1 mM, 15 mL of PP-ol solution, 15 mg of catalyst, UV, 6 h, 30 °C	This study
TiO_2_ US22-B	57	10.1	42	20	3	–	1 mM, 15 mL of PP-ol solution, 15 mg of catalyst, UV, 6 h, 30 °C	This study

One of the most important feature in heterogeneous catalysis is the stability and potential reusability of the solid catalyst keeping the efficiency high upon multiple-uses. To study the reusability photocatalytic ability of the synthesized titania samples, tests were performed according to the details presented in the experimental section, with the obtained results to be presented in Figure S8**.** MagS samples showed a declining trend of PP-ol conversion even after the second run. On the contrary, US22-B sample revealed a minimal decrement in PP-ol conversion performance, with the selectivity and yield towards the targeted compound to be almost unchanged even up to 5 cycles.

Expect the stable photoreactivity, it is essential to avoid the Ti leaching after experiments. The X-ray fluorescence (XRF) of the solution after the fifth run of photocatalytic activity of all samples was also performed in order to study the leaching of Ti during/after the photocatalytic experiment. The results (Figure S9) did not show any peak corresponding to Ti metal, which suggested that no leaching was occurred and that all the samples were stable after the fifth cycles of photocatalytic experiments. So, these results suggest that the ultrasound synthesized sample US22-B can be used for multiple cycles of photocatalytic experiments, since it showed an almost unchanged high photocatalytic performance and it is stable after the photocatalytic experiments.

### 3.4 Effect of calcination on the photo-activity

**Fig. 5:**
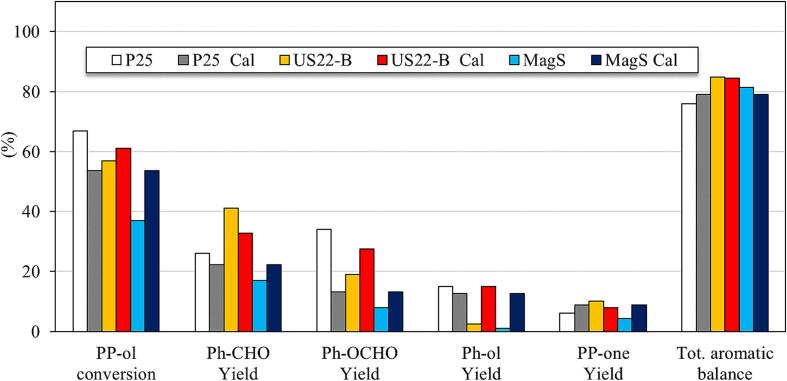
Effect of calcination on photocatalytic activity for the oxidation of 2-phenoxy-1-phenylethanol to the corresponding products by using commercial P25, US22-B and MagS samples.

The US assisted titania sample (US22-B), along with MagS and P25 was calcined at 500 °C for 5 h in order to study the effect of calcination on the physicochemical features, more importantly on crystallinity, and as a result on the photocatalytic selective performance. The calcined MagS and US22-B samples showed the presence of anatase phase (Figure S10, Table S1), whereas no effect of calcination was found on the crystallinity of the commercial P25, as it was expected since this material is commercial synthesized at higher temperature. The photocatalytic performance of these calcined samples was studied at the same experimental conditions for the partial selective conversion of PP-ol to the corresponding products. The results (Figure 5) showed that for P25 PP-ol conversion and yield of the crucial monoaromatics were significantly lower, suggesting that the presence of pre-adsorbed humidity play a key role on its photoreactivity. The calcination in the case of US22-B had a small positive impact on PP-ol conversion and Ph-OCHO formation, but a slightly negative effect towards formation of Ph-CHO. The calcined MagS (MagS-Cal) sample possessed higher conversion of PP-ol and also slightly higher yield of all the corresponding products as compared to the not-calcined MagS sample, but not higher comparing to US22-B. It can be concluded bearing in mind all the above, that our novel US-assisted 1-D nanotitanate sample has the best performance, even avoiding the energy-consuming step of calcination that is crucial for the synthesis of a plethora of other showcased TiO_x_ materials, without a discount on stability and reusability.

### 3.5 Sonophotocatalytic oxidation of 2-phenoxy-1-phenylethanol

**Fig. 6:**
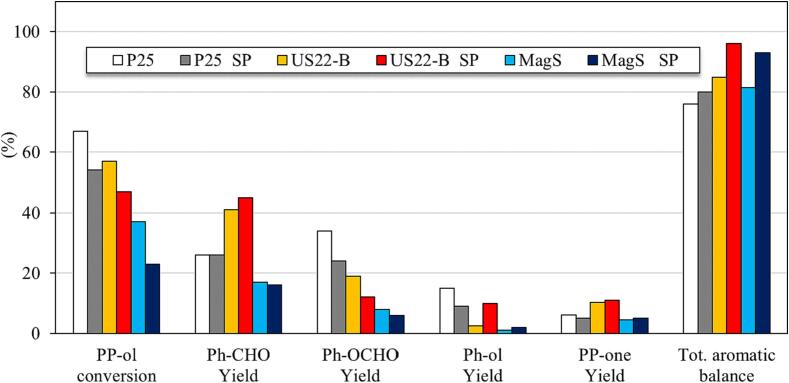
Sonophotocatalytic activity for the selective conversion of PP-ol to the corresponding products using MagS, US22-B and P25 samples (The use of SP is linked to the sonophotocatatytic experiments).

The use of ultra-sonication in photocatalysis, as a process intensification tool, known as sonophotocatalysis, has been widely investigated for the degradation of organic pollutants [46]. On the contrary, sonophotocatalytic applications for selective synthetic processes are few. One of the most crucial aspect is the design and development of the sonophotoreactor and optimization of various parameters, as we showcased previously [47]. In this work, the catalytic experiments were performed by the applying sonication during the photocatalytic experiments, replacing also the magnetic stirring taking advantage of the physical effects derived upon sonication. The results obtained from the sonophotocatalytic experiments are compared with the results of photocatalytic experiments up to 6 h of UV light irradiation in Figure 6.

P25 sample showed an lowered of PP-ol conversion (64 %) in sonophotocatalytic experiments as compared to photocatalytic experiments (52 %), with the yields of Ph-OCHO, Ph-ol and PP-one to be also a bit lower in sonophotocatalysis than photocatalysis. The yield of Ph-CHO was found although unchanged, but the aromatic balance was found slightly enhanced. In the case of US22-B, the sonophotocatalytic PP-ol conversion (47 %) was only slightly lower comparing to the photocatalytic test, but the yields of the monoaromatics except Ph-ol were slightly increased compared to photo-catalysis. The most important outcome of the sonophotocatalytic tests was the achieved by US22-B ultimately high aromatic balance of 97 %, a value much higher than that achieved in the photocatalytic experiments (84 %). MagS sample showed the lowest PP-ol conversion (23 %) in the case of sonophotocatalysis, a value lower by 35 % compared to the photocatalytic activity under magnetic stirring. Although, the yield of all the products was found to be almost the same in photocatalytic and sonophotocatalytic tests, and as a result the aromatic balance to be improved upon utilization of ultrasonication

These results revealed that the coupling of sonication with photocatalysis can have positive effect on selective redox and bond cleavage reactions, since the yields of most of the products were found equal or higher upon sonication. Although, the conversion of the PP-ol was found to be slightly lower in the case of sonophotocatalysis comparing to photocatalysis under magnetic stirring, the yield of the products, especially the targeted product such as Ph-CHO was interestingly either equal or higher than the yield of Ph-CHO obtained by photocatalysis. These results suggest a lowering in the formation of side products, such as undesired aliphatic compounds. Due to this, the selectivity toward the aromatic compounds was improved, leading to an enhanced aromatic balance for the sonophotocatalytic tests compared to the photocatalytic tests. Eliminating the formation of small in molecular weight aliphatic compounds has a positive effect in order to avoid purification steps.

The enhanced selectivity can be linked to the chemical and physical effects arisen upon ultrasound irradiation, such as the better mixing effect and mass transfer, enhanced de-aggregation, the formation of hot-spots etc. The differences in suspension mixing during photocatalysis and sonophotocatalysis can in general affect the selective oxidation process, as we also showed previously [47]. During the magnetic stirring, the nanoparticles in the suspension may be remained aggregated and moved as a chuck, leading to a less selective reaction. Ultrasound irradiation can improve the deaggregation and deagglomeration, which can lead to an increase in the surface area and hence in the accessibility of the catalytic sites [47], [48].

Sonication solely can catalytically degrade various organic compounds, such as such paracetamol, rhodamine, congo red, reactive blue, 4 methyl orange, methylene blue etc., in the aqueous media [5]. Eventhough acetonitrile was used as the medium/solvent of the reaction in the present study, to explore the potential sonolytic as well as sonophotolytic decomposition of PP-ol was essential as control experiments, as it was to perform the heterogeneous sonocataytic tests for P25, US22-B, and MagS. The results (Figure S11a,b) showed that no conversion of PP-ol was showcased either for the sonolytic, photolytic, sonophotolytic, and for the sonocatalytic experiments. These results are also in good agreement with the sonocatalytic results of P25 for the degradation of 2-dibenzothiophenes in acetonitrile using TiO_2_ P25 and confirmed that no reaction occurred with ultrasound (45 kHz, 50 W) irradiation alone, i.e. in the absence of TiO_2_ P25 or light (200 W Hg–Xe lamp), but under sonophotocatalytic conditions, degradation of 2-dibenzothiophenes was observed [48].

### 3.6 Photocatalytic mechanistic insights

**Scheme 2:**
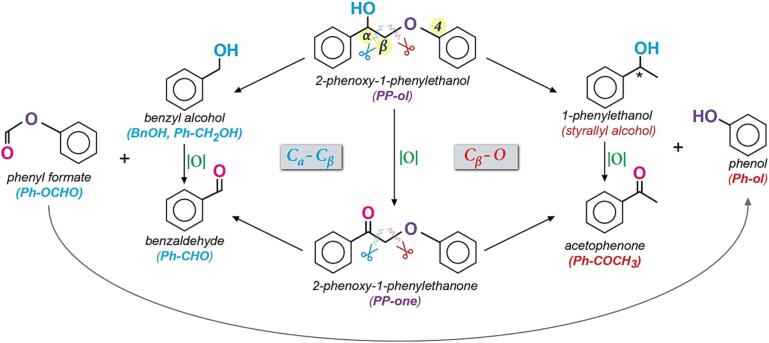
All the possible reaction pathways for the depolymerization and oxidation of *β*-O-4 linkage of the lignin-inspired model compound 2-phenoxy-1-phenylethanol (PP-ol) towards the formation of the desired monoaromatics.

To gain insights regarding the potential origin of the formed products from the (sono)photocatalytic depolymerization or/and oxidation of PP-ol or the derived aromatics, a series of reactions pathways can be involved. The cleavage of the β-Ο-4 linkage can undergo via the break of the C_β_–O or C_α_– C_β_ leading to different compounds as can be observed in Scheme 2. It is also possible PP-ol to be partially oxidized to PP-one, and PP-one to breakdown to compounds that can be received after the partial oxidation of the monoaromatics formed by PP-ol decomposition [1], [2], [11]. In our case, the scenario of PP-ol partial oxidation should be excluded, since only limited amount of PP-one was detected and bearing in mind that the photocatalytic tests revealed that PP-one is not converted toward the monoaromatic compounds by our photocatalysts under the herein followed reaction protocols (Figure S12). Hence, the monoaromatic compounds such as Ph-CHO and Ph-OCHO are formed by the catalytic valorization depolymerization and/or oxidation) of PP-ol.

Photocatalytic studies using pure Ph-OCHO solutions showed that Ph-OCHO is converted to a significant amount of Ph-ol (Figure S13). Another interesting observation was found that the Ph-OCHO was converted to the Ph-ol during the adsorption–desorption equilibration step for all the herein studied samples without light irradiation (Figure S14a, b). In addition, the photolysis studies of Ph-OCHO did not show any conversion (Figure S14c). So these results suggested that Ph-OCHO is the potential origin of the detected of Ph-ol. In addition, the no detection of acetophenone or/and styrallyl alcohol (1-phenylethanol) strongly support that the photocatalytic decomposition of the *β-Ο-4* linkages undergo selectively via the cleavage of the C_α_–C_β_ bond. Considering the higher conversion and formation rate/extend of PP-ol, BnOH, Ph-OCHO, and Ph-ol as well as the lower selective and yield towards the desired monoaromatics as presented in this work, it is obvious that the synthesized samples and especially the ultrasound-derived one can be regarded as a prosperous nanomaterial to be utilized as photocatalyst for the selective upgrade of lignin.

### 3.7 Photocatalytic studies of the catalysts for the conversion of PP-ol by the addition of various scavengers

Photoinduced charges such as holes, electrons, or reactive oxygen species (ROSs) like hydroxyl (•OH) and superoxide (O_2_^–•^) free radicals play a key role in photocatalysis [49], and hence to determine which ones are responsible for the elevated photocatalytic activity of our photocatalysts was an ultimate important aspect. Towards this direction, the addition of specific compound that acts as scavenger of a specific reactive specie is a strategy to study to analyze the involved mechanisms [50], [51]. It is crucial to make loud and clear as it was aforementioned that this work is the first one in which designed and synthesized novel titania based nanomaterials for the selective photocatalytic valorization/depolymerization of a lignin-inspired model compound containing a β-Ο-4 linkage. And in general, only a very limited amount of works exist for the photocatalytic cleavage of β-Ο-4. For example, Liang et al. [8], proposed three potential mechanisms for the photocatalytic selective cleavage of C-C bond of the PP-ol molecule which are catalyzed from the photogenerated hole and the singlet oxygen as concluded based on scavengers tests. In another researcher by Han et al. using an ultrathin Metal/CdS material [7], it was showed that photogenerated holes play a key role in the photocatalytic decomposition and/or oxidation of PP-ol compound to the corresponding products.

Zhao et. al. [3] reported a possible mechanism for the conversion of PP-ol to the corresponding products using Au–Pd nanoparticles supported on covalent triazine frameworks. Initially, PP-ol was converted to 2-phenoxyacetophenone followed by the conversion to the corresponding peroxide. The formation of phenyl formate and benzoic acid was occurred by the cleavage of the C-C bond and the peroxide. At the same time, the C-O bond was also cleavage to produce the phenol and the phenylglyoxal. Further, phenyl formate is converted to phenol, while phenylglyoxal was converted to methyl benzoate, methyl formate and benzoic acid. In another work by Cui et. al. [4] it was proposed the mechanism of the PP-ol conversion to the detected products using atomically dispersed Pt_1_/N-CNTs through electro-catalysis. Firstly, the *tert*-butyl hydroperoxide produced *tert*-butoxyl radical and *tert*-butyl peroxyl radical, and C_β_ intermediate radicals is formed by the C_β_-H cleavage of the PP-ol on the Pt_1_/N-CNTs anode surface. The *tert*-butoxyl radical and *tert*-butyl peroxyl radical react with the C_β_ intermediate radicals to form the intermediate complex by the radical/radical cross-coupling reaction, which initiates the cleavage of the C_α_ − C_β_ and the formation of aromatic aldehyde, phenol, and CO_2._ The products obtained, such as Ph-ChO, Ph-OCHO, and Ph-ol by the catalytic conversion of PP-ol, differ from the herein reported one. According to our literature survey, no potential mechanism was reported for the formation of all detected in our study products. The identification of the active species for the catalytic reaction is necessary in order to understand the potential mechanism of the reaction.

Since no report exist regarding the mechanistic insights for PP-ol decomposition using titania, we performed scavenger experiments in order to study and identify the main active species that can be responsible for the photocatalytic selective cleavage of C-C bond of the PP-ol. The utilized scavenger compounds are oxalic acid (OA) and potassium iodide (KI) were used as h^+^ scavengers, silver nitrate (Ag) as e^-^ scavenger, 1,4-Benzoquinone (BQ) as O_2_^•-^ scavenger, and *tert*-butanol (t-BtOH) as ^•^OH scavenger. The initial concentration of the scavenger substance per initial concentration (1.5 mM) of PP-ol substrate was kept equimolar (1:1).

**Fig. 7:**
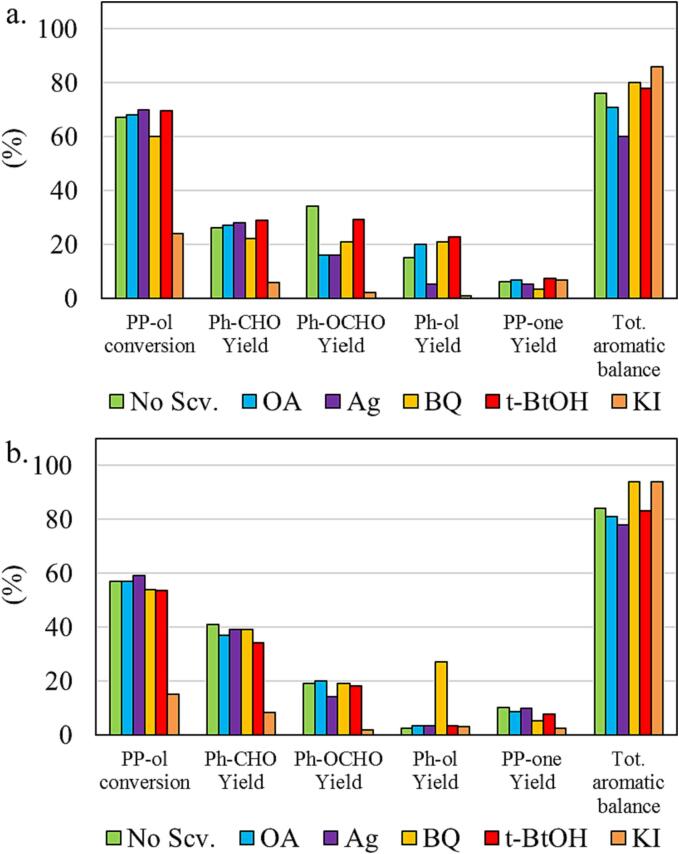
Photocatalytic study of P25 (a), and US22-B (b) samples by the addition of scavengers for the various species.

The results (Figure 7) in the cased of the commercial benchmark photocatalyst TiO_2_ P25 revealed that the addition of the organic h^+^ scavenger (OA) did not affect the PP-ol conversion but the use of the inorganic scavenger had a negative impact on PP-ol conversion. Based on these outcomes we can conclude that indeed holes are responsible for the decomposition of PP-ol, but the use of OA is not a right scavenger for this kind of materials. The predominant role on PP-ol conversion can be further supported by the fact that scavenging of all the other possible reactive species (e^-^, ^•^OH, O_2_^•-^) did not affect the extend of PP-ol conversion. On the regards of the formed products and taking a look on how their yields were impacted upon KI addition, it is obvious that the yields of the all the monoaromatics as well as of PP-one were demised. In order to extract specific conclusions for the reasons behind this effect, it is crucial to consider the effect of electron scavenger addition. The yield of Ph-CHO was not changed. Although, the yield of Ph-OCHO was half and of Ph-OH zero. Considering all the above, it can be assumes that both holes and electrons are involved in the Ph-OCHO conversion to Ph-OH or decomposition, with the free electrons to be more responsible.

In the case of the synthesized and best performing photocatalyst US22-B, only holes scavenging affected negatively the PP-ol conversion and hence the yields of the desired monoaromatics. This is a good agreement with the high selective photocatalytic reactivity which can be linked with the conclusive involvement of the holes. Since we have aromatics compounds, it is very crucial the thermodynamics and the orientation of the aromatic part adsorption on the surface. We had showed previously the presence of the surface hydroxyl groups showcased different kind of interaction with the benzyl alcohol and benzaldehyde leading to a more selective partial photo-oxidation efficiency [51]. This can be supported from the results using benzoquinone as scavenger. As in the case of TiO_2_ no alterations on the photoreactivity were observed, for US22-B, the addition of BQ led to two dramatically increment of the Ph-OH yield, with all the rest yields to remain intact. The conversion of BQ can be the source of the Ph-OH formation, which happen only in case of the synthesized sample due to uniqueness of surface interactions. That can be additionally supported from the analogues results recorded for the other synthesized samples, MagS (Figure S15).

**Scheme 3:**
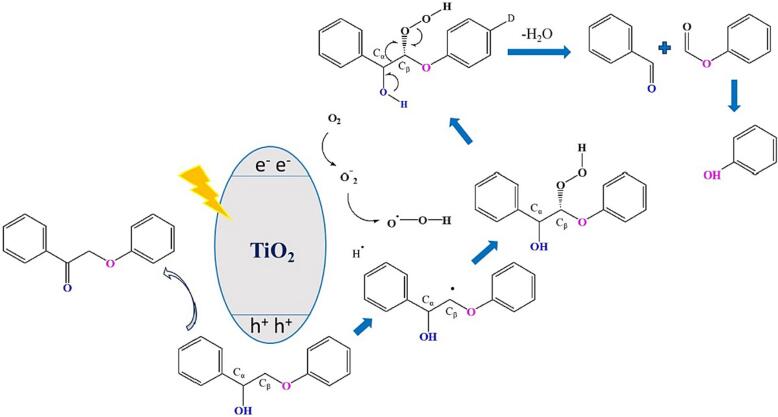
Proposed potential mechanism for the catalytic cleavage of C_α_–C_β_ bond of PP-ol.

Since the predominant obtained products of the decomposition of PP-ol were Ph-CHO and Ph-OCHO, and considering that holes were the majorly involved active species, a potential mechanism is proposed in Scheme 3. At the first step, C_β_ undergoes deprotonation upon attack from a hole. The formed diaromatic radical reacts with the hydrogen superoxide radical (HOO^•^) resulted from the dissolved molecular oxygen, forming an intermediate complex. Intermolecular electron rearrangement leads to electron transfer from the hydroxyl group to the C_α_ and lead to the cleavage of C_α_-C_β_ bond and O-O bond of C_β_ results in the formation of benzyl aldehyde and phenyl formate. The minimal detected amount of phenοl can be due to the redox photoconversion of phenyl formate.

As above described the physicochemical properties and the results of catalytic activity can be summarised as the proper designing and synthesis of the nanomaterials for the selective catalytic oxidation of lignin-inspired model compounds is very important. The utilization of ultrasound with the optimized power during the synthesis of nanomaterials is considered a prosperous approach for the designing of a proper catalyst. The utilization of low frequency in the range of sonochemistry, such as 22 kHz, mostly led to the physical effect of ultrasound, which can improve the growth of nanostructure. The higher catalytic selective conversion of lignin inspired model molecule by using US22-B samples to the targeted products such as PhCHO is due to many possible factors. The first factors the US-assisted synthesized nanomaterials possessed the highest textural properties, such as higher specific surface area and pore size distribution of nanomaterials. The enhanced textural properties as well as morphology of the US assisted sample, especially the observation of interlayer spacing, is also a curcial aspect which helps to improve the absorption of the lignin inspired model compounds, and also it enhances the light penetration inside the samples. Another, the surface heterogeneity of the US-assisted nanomaterials showed the existence of the basic medium, which confirms the blockage of acidic groups on the surface. Another surface composition of the US-assisted samples by the XPS analysis also revealed the presence of sodium on the surface, which also enhanced the basicity of the surface. The presence of the sodium on the surface of the synthesized photocatalyst measured by the XPS analysis improved the surface's basicity. Moreover, the intensely elevated ratio of the oxygen to the titanium atom indicated the occurrence of hydroxyl radicals between the layers of the 1-D nanostructures, which is in good agreement with the thermal analysis as well as the previously reported literature [24], [31], [50]. The elevated amount of carbon-containing functional groups such as carbonyl and carboxyl on the surface can also positively impact the catalytic performance of the titania-based nanostructures [31], [51], [52]. The higher oxygen species corresponding to C = O on the surface of US22-B sample, as described by XPS analysis, also suggested that the C = O functional group may lead to the photosinzitizer effect, which improved the light penetration [53], [54], [55]. As XRD analysis described the US22-B sample possessed a predominentaly ambiguous phase with a small size anatase crystal phase, which also improved the catalytic activity compared to well-defined crystal phase [56]. Another higher hydroxyl radicals by the thermal analysis also improves the catalytic activity [37].

## 4 Conclusions

In summary, we showcased a green approach using ultrasonication as a synthesis tool for the development of novel semiconductor nanophotocatalysts, avoiding the use of noble or rare elements and harmful chemical and in addition avoiding the energy-demanding step of calcination at high temperature. The utilization of low-power low-frequency (22 kHz) ultrasound irradiation led to nanostructures with elevated textural features and surface chemical heterogeneity compared to the non-US/silent synthesized sample and the commercial benchmark TiO_2_ P25 nanoparticles. After optimization of the US power, the best US derived sample, US22-B, showed the highest selectivity and yield of benzyl aldehyde formation by selective partial photo-catalytic oxidation of benzyl alcohol, compared to all other ultrasound-assisted synthesized sample, as well as in silent and P25. Even more importantly, US22-B showed to be very efficient for the selective valorization of lignin-inspired model compound 2-phenoxy-1-phenylethanol (PP-ol). The selective nature of controlled conversion was based on the photocatalytic cleavage of C_α_–C_β_ bond of *β-O-4* linkage, leading almost predominately to the value-add compounds benzyl formate and benzyl alcohol. Going a step ahead, sonication was adapted simultaneously with light irradiation for the first time in order to explore the sonophotocatalytic valorization of a lignin-inspired diaromatic model compound. The coupling of two kinds of powers, sono and photo, not only led to the abandonment of the mechanical magnetic stirring, but it had a positive synergistic effect on the selective conversion of PP-ol to the desired compounds, with the aromatic balance to retained almost intact (97 %). In the case of sonophotocatalytic experiments, US22-B revealed the greatest Ph-CHO yield and highest aromatic balance. All in all, it can be concluded that the use of ultrasound for the design of novel nanocatalysts for the use towards sonophotocatalytic selective lignin depolymerization to value-added platform compounds can be assumed as an effective and prosperous strategy.

## CRediT authorship contribution statement

**Abdul Qayyum:** Writing – review & editing, Writing – original draft, Visualization, Methodology, Investigation, Formal analysis, Data curation, Conceptualization. **Dimitrios A. Giannakoudakis:** Writing – review & editing, Writing – original draft, Conceptualization. **Dariusz Łomot:** Visualization, Investigation, Formal analysis, Conceptualization. **Ramón Fernando Colmenares-Quintero:** Writing – review & editing, Funding acquisition. **Kostiantyn Nikiforow:** Investigation, Formal analysis. **Alec P. LaGrow:** Investigation, Formal analysis. **Juan Carlos Colmenares:** Writing – review & editing, Conceptualization, Resources, Project administration.

## Declaration of competing interest

The authors declare that they have no known competing financial interests or personal relationships that could have appeared to influence the work reported in this paper.

## Data Availability

No data was used for the research described in the article.
