# Supplementary material for: Selective (sono)photocatalytic cleavage of lignin-inspired β–O–4 linkages to phenolics by ultrasound derived 1-D titania nanomaterials
Source: Ultrason Sonochem. 2024 Mar 2;104:106829. doi: 10.1016/j.ultsonch.2024.106829 (PMC10937310; doi:10.1016/j.ultsonch.2024.106829)
Supplement: Supplementary data 1 [file mmc1.docx]

**SUPPLEMENTARY INFORMATION**

**Selective (sono)photocatalytic cleavage of lignin-inspired *β*–O–4 linkages to phenolics by ultrasound derived 1-D titania nanomaterials**

**﻿**Abdul Qayyum^a,^*, Dimitrios A. Giannakoudakis^a,b,^*, Dariusz Łomot^a^, Ramón Fernando Colmenares-Quintero^c,^*,  Kostiantyn Nikiforow^a^, Alec P. LaGrow^d^, Juan Carlos Colmenares^a,c,^*

*^a^ Institute of Physical Chemistry, Polish Academy of Sciences, Kasprzaka 44/52, 01-224 Warsaw, Poland.*

*^b^ Laboratory of Chemical and Environmental Technology, Division of Chemical Technology, Department of Chemistry, Aristotle University of Thessaloniki, Thessaloniki, GR-541 24, Greece.*

*^c^ Engineering Research Institute (In^3^), Universidad Cooperativa de Colombia, Medellín 50031, Colombia*

*^d^Scientific Imaging Section, Okinawa Institute of Science and Technology Graduate University, Kunigami-gun, Okinawa 904-0412, Japan.*


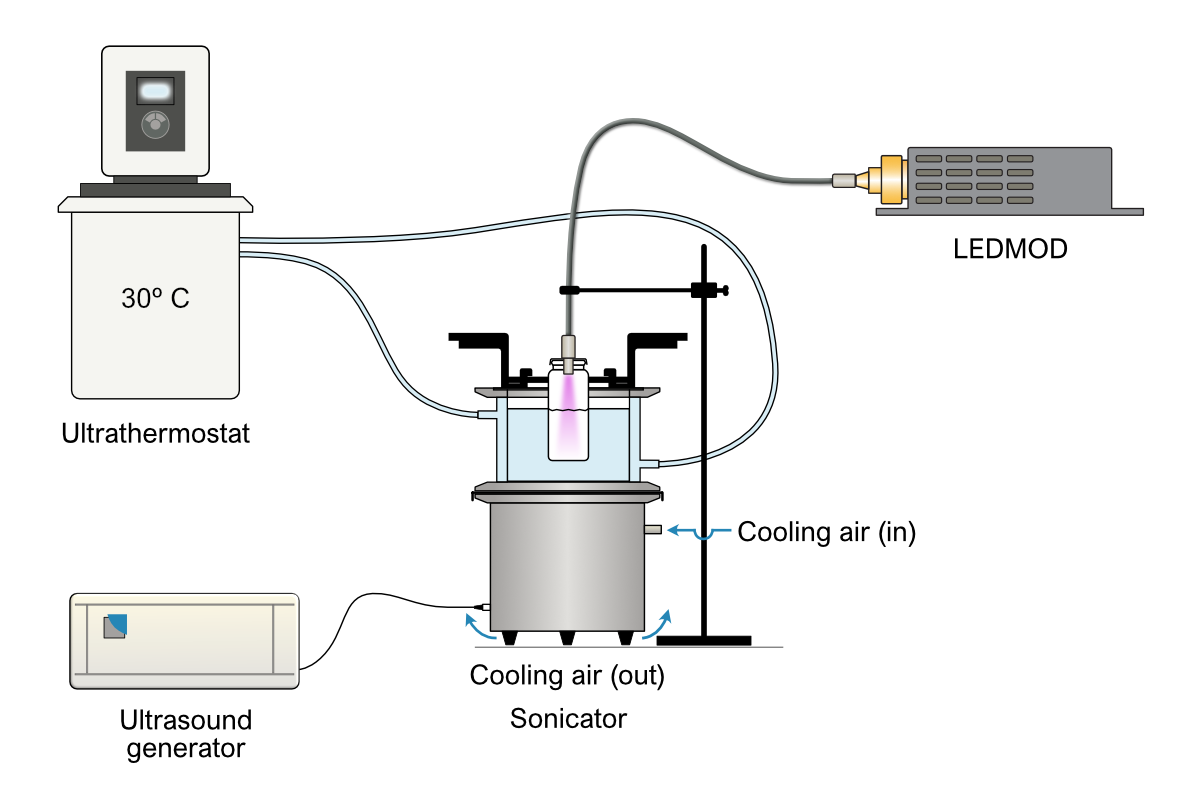


**Scheme S1.** Sono-photocatalytic setup.

**Table S1.** Calculated by Scherer method values of crystallite size (in nanometers).

| **Sample** | **Dcr Anatase (011) [nm]** | **Dcr Rutile (110) [nm]** |
| --- | --- | --- |
| P25 | 17.3 | 22.3 |
| MagS | - | - |
| US22-A | - | - |
| US22-B | 3.1 | - |
| US22-C | - | - |
| US22-D | - | - |
| US22-E | - | - |
| P25 Cal | 17.4 | 22.5 |
| MagS Cal | 9.1 | n/a |
| US22-B Cal | 9.5 | n/a |


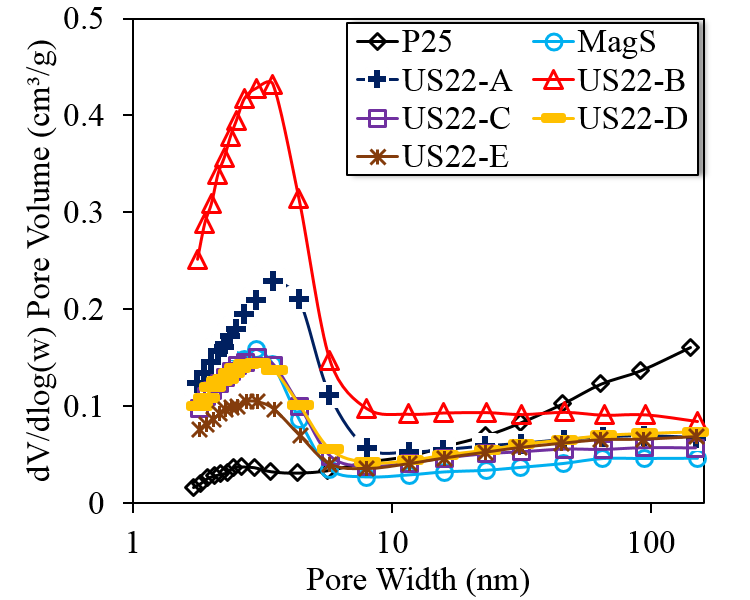


**Figure S1.** Pore size distribution based on BJH method for P25, MagS and US assisted synthesis samples by 22 kHz with different powers.


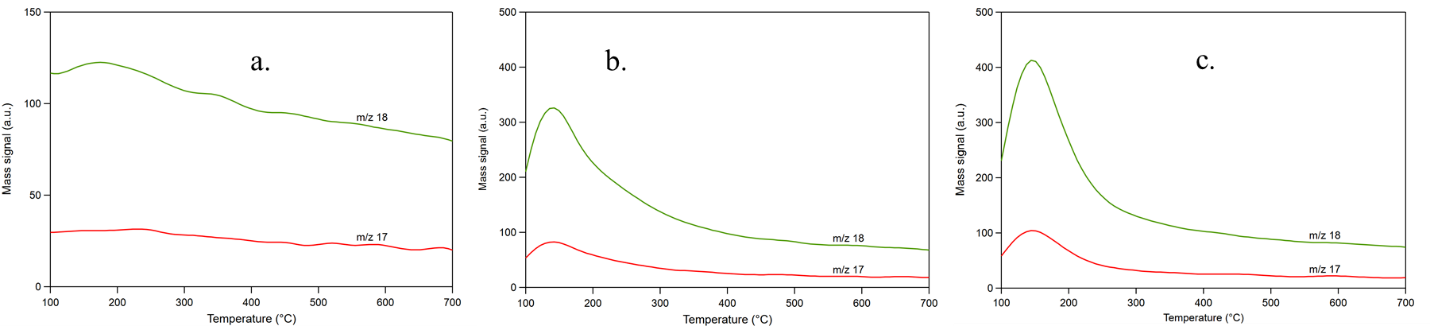


**Figure S2.** Temperature program desorption (TPD) in range 1–100 m/z of P25 (a), US22-B (b), MagS (c).


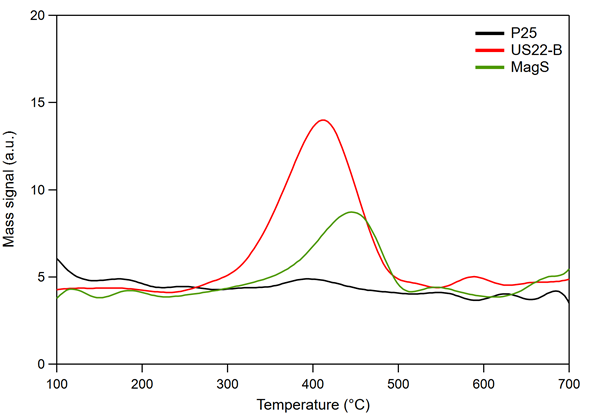


**Figure S3.** Temperature program oxidation of samples after TPD measurement for m/z 44 (CO_2_).


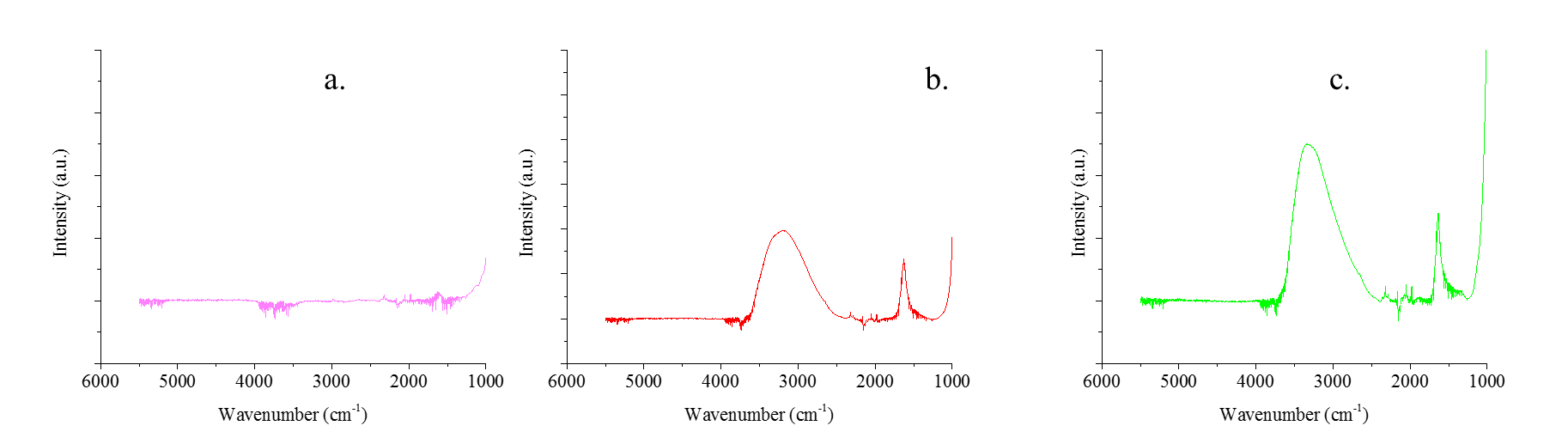


**Figure S4.** Fourier transform infrared measurement for P25 (a), US22-B (b), MagS (c).


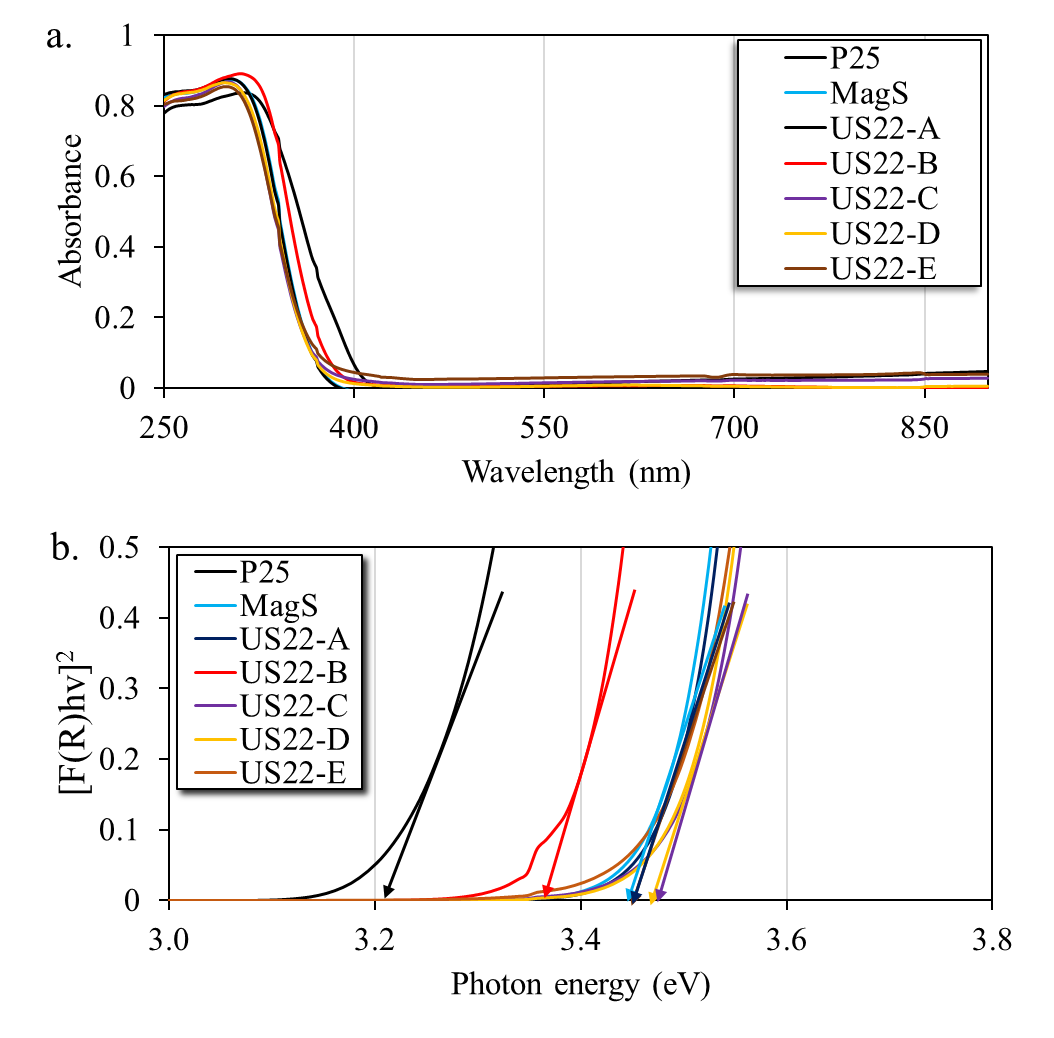


**Figure S5.** (a) Diffuse reflectance absorption spectra (b) Tauc plots for the P25, MagS and US assisted synthesized TiO_2_ by low amplitude; (c) Diffuse reflectance absorption spectra (d) Tauc plots for the P25, MagS and US assisted synthesized TiO_2_ by high amplitude.

**
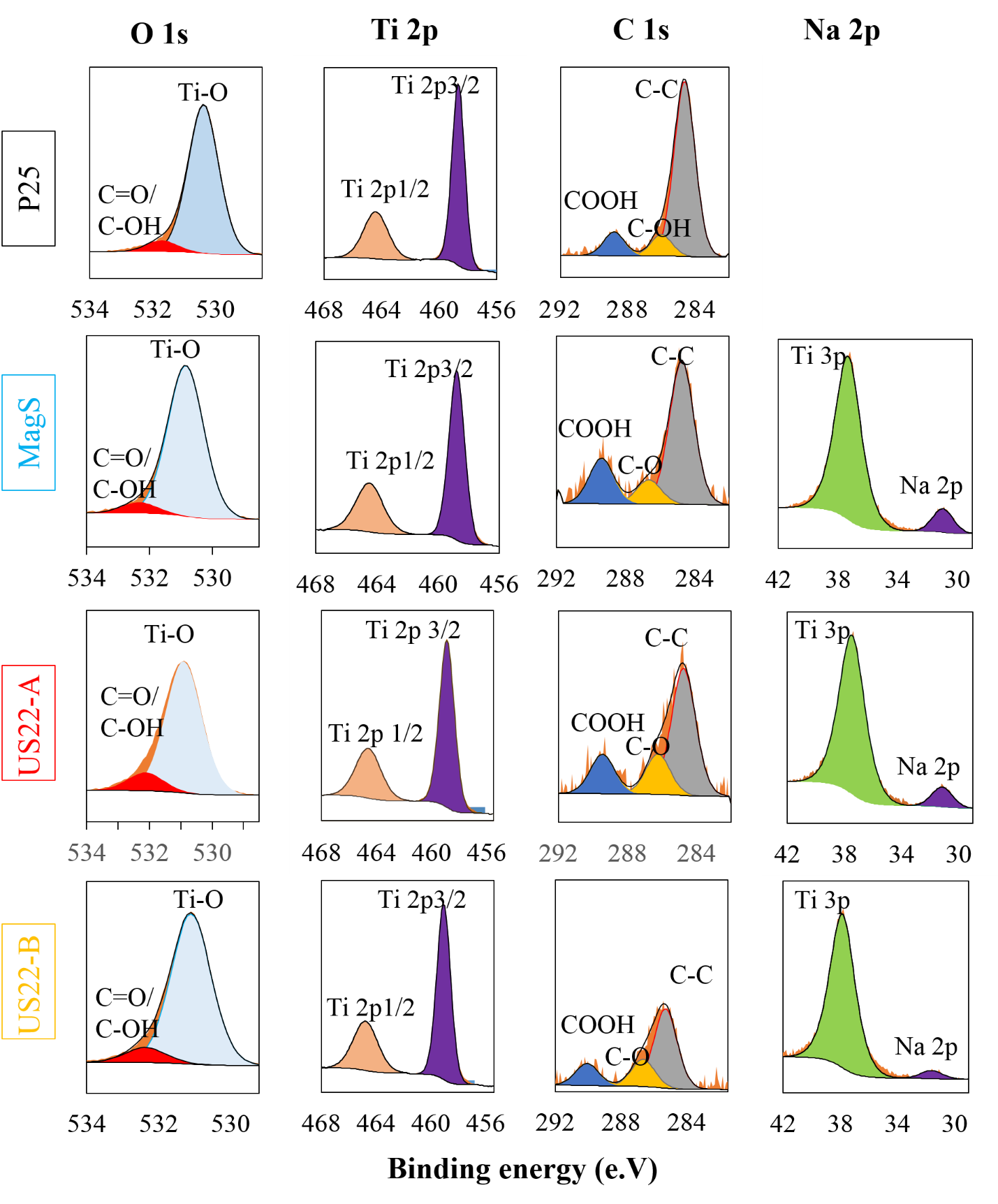
**

**Figure 6.** Deconvoluted high-resolution core energy level XPS spectra for P25, MagS, US22-A, and US22-B samples

**
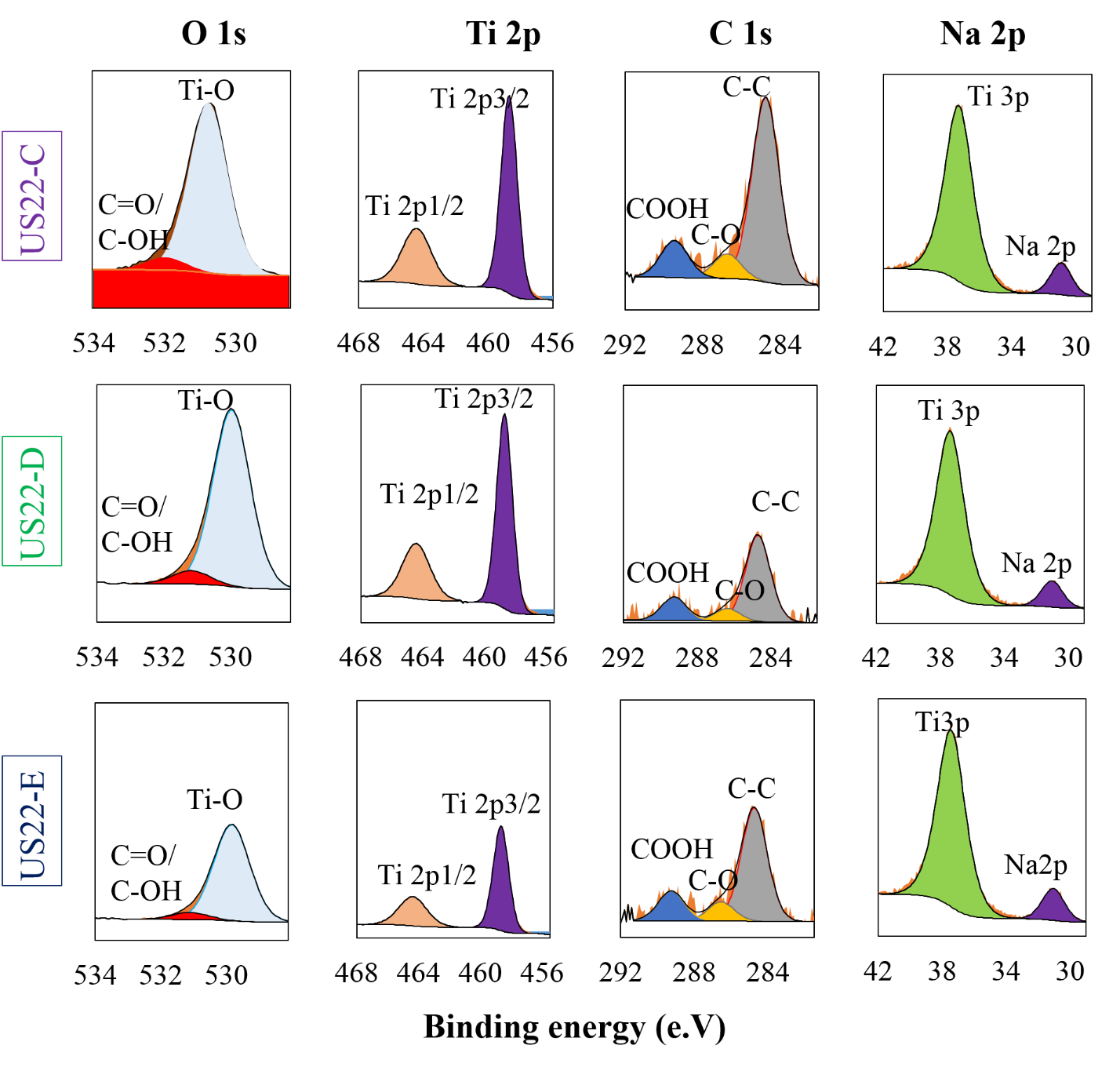
Figure S7.** Deconvoluted high-resolution core energy level XPS spectra for US22-C , US22-D and US22-E samples.


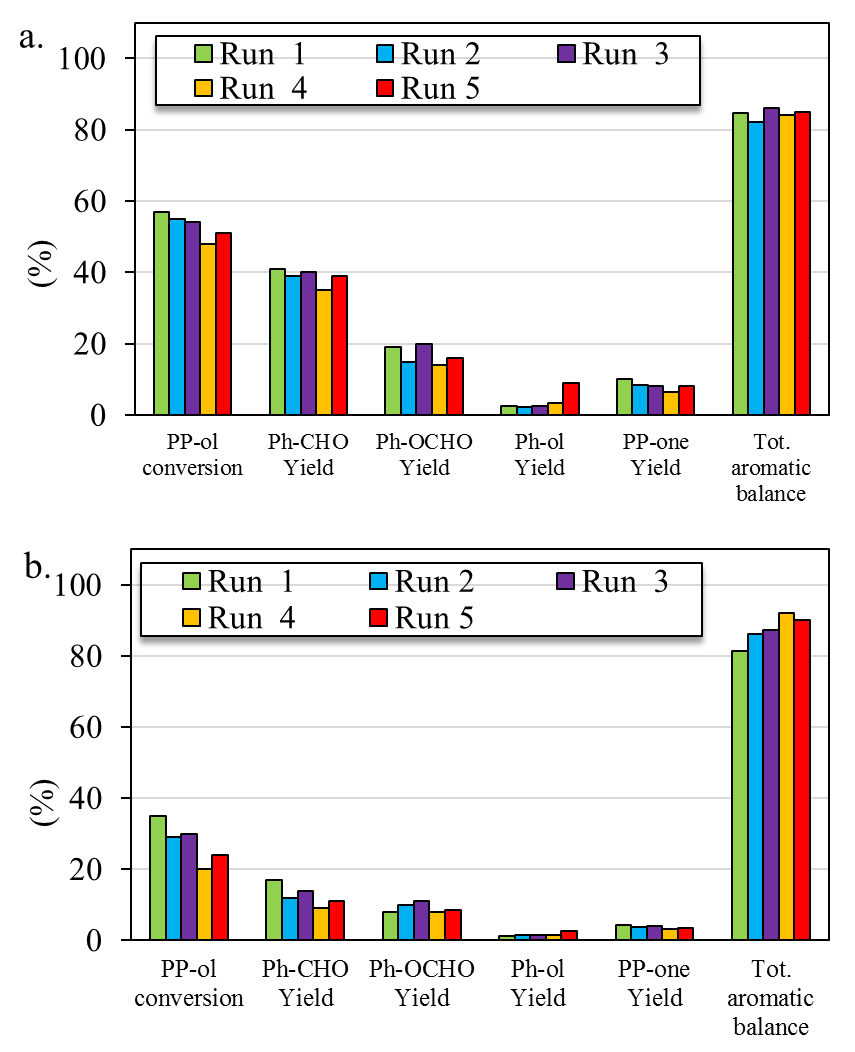


**Figure S8.** Reusability photocatalytic studies of commercial US22- B (a), MagS (b) samples up to 6 h of light irradiation.

**
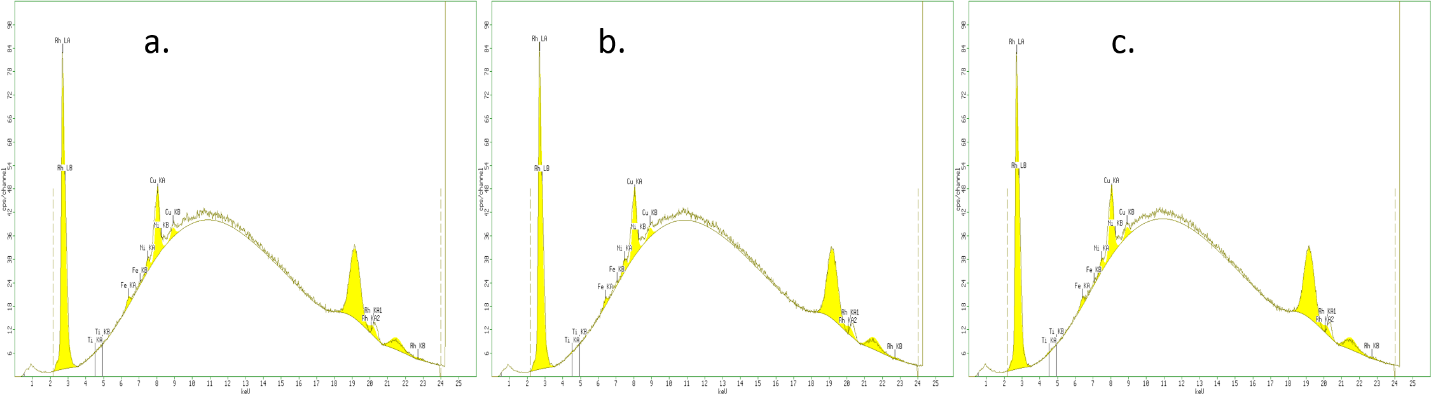
**

**Figure S9.** XRF spectra of the solution of after the 5^th^ run of photocatalytic experiments for the conversion of PP-ol substrate for P25 (a), US22-B (b), MagS (c).

**
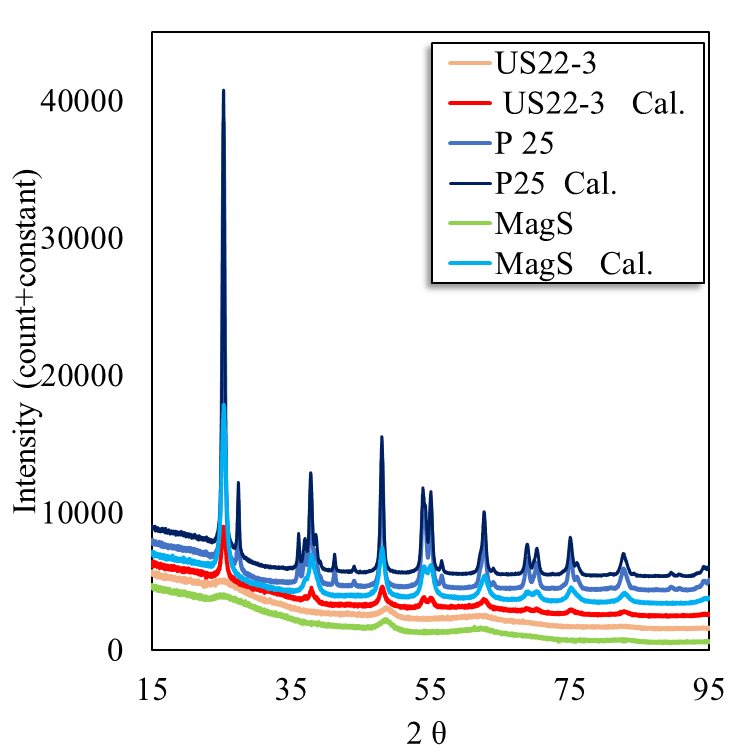
**

**Figure S10.** Effect of calcination on the crystallinity of the TiO_2_ samples


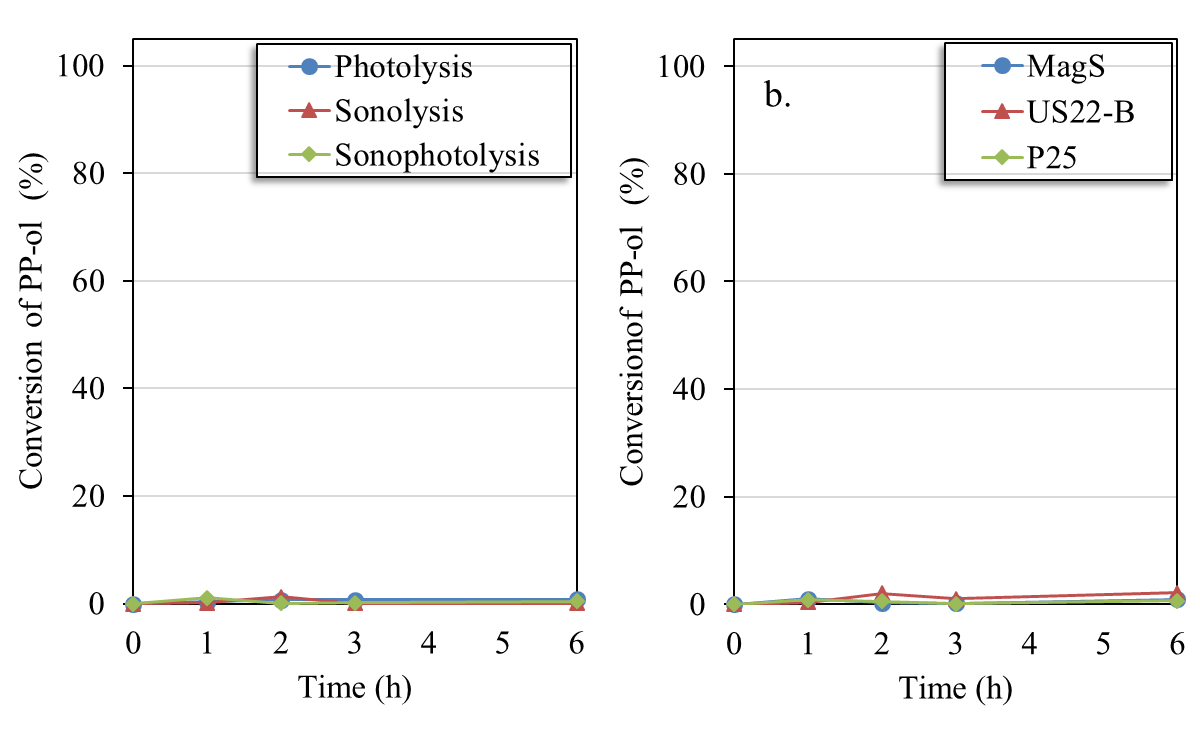


**Figure S11.** Control experiments for the MagS, US22-B and P25 samples for the conversions of PP-ol by photolysis, sonolysis and sonophotolysis (a), sonocatalysis activity (b).


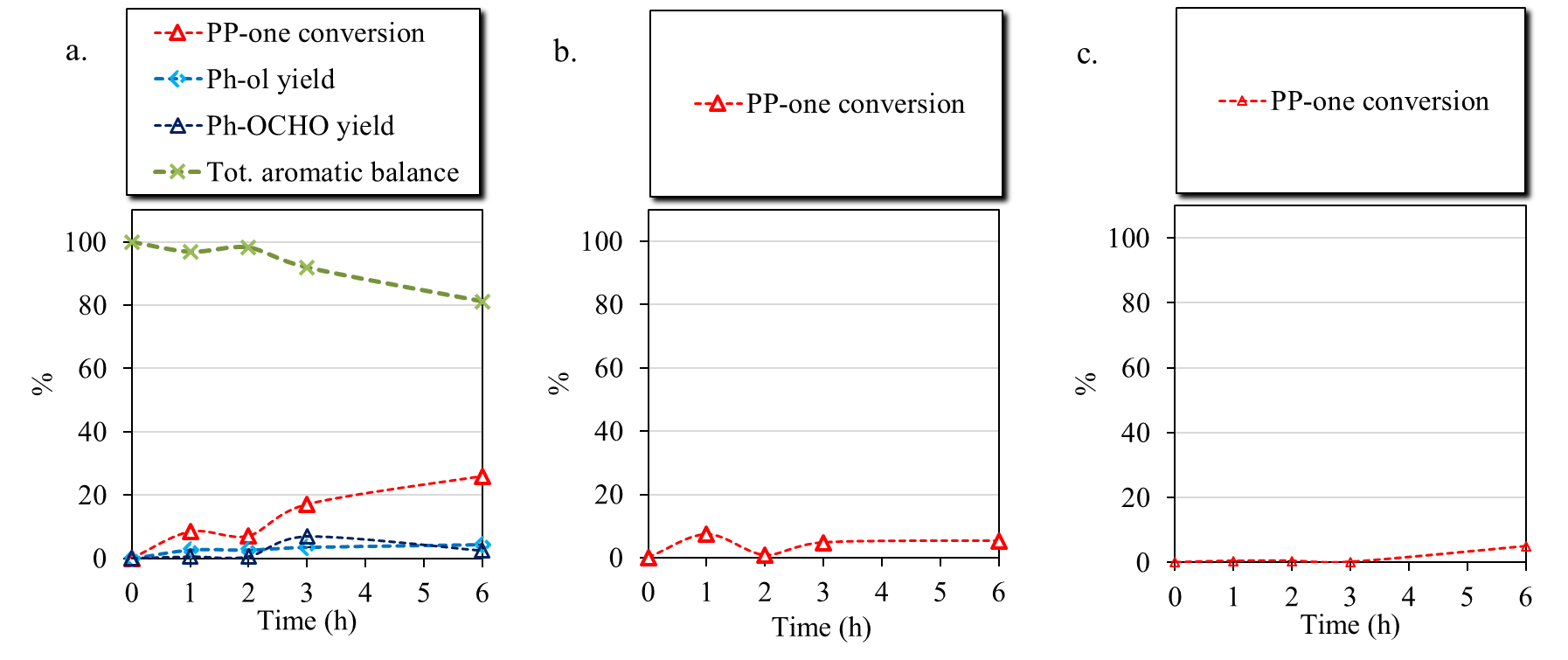


**Figure S12.** Photocatalytic conversion of 2-phenoxy-1-phenylethanone by using samples MagS (a), US22-B (b) and P25 (c).


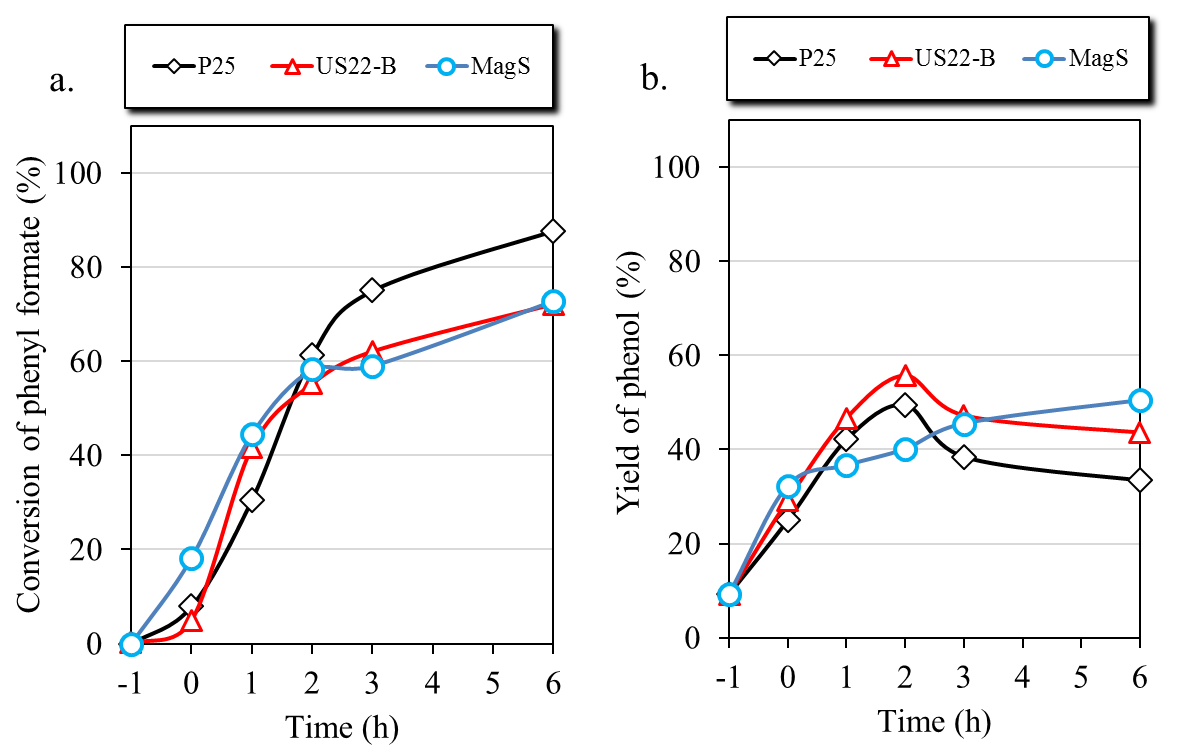


**Figure S13.** Photocatalytic activity of commercial and synthesized samples for the conversion of phenyl formate (a), yield of Phenol (b).


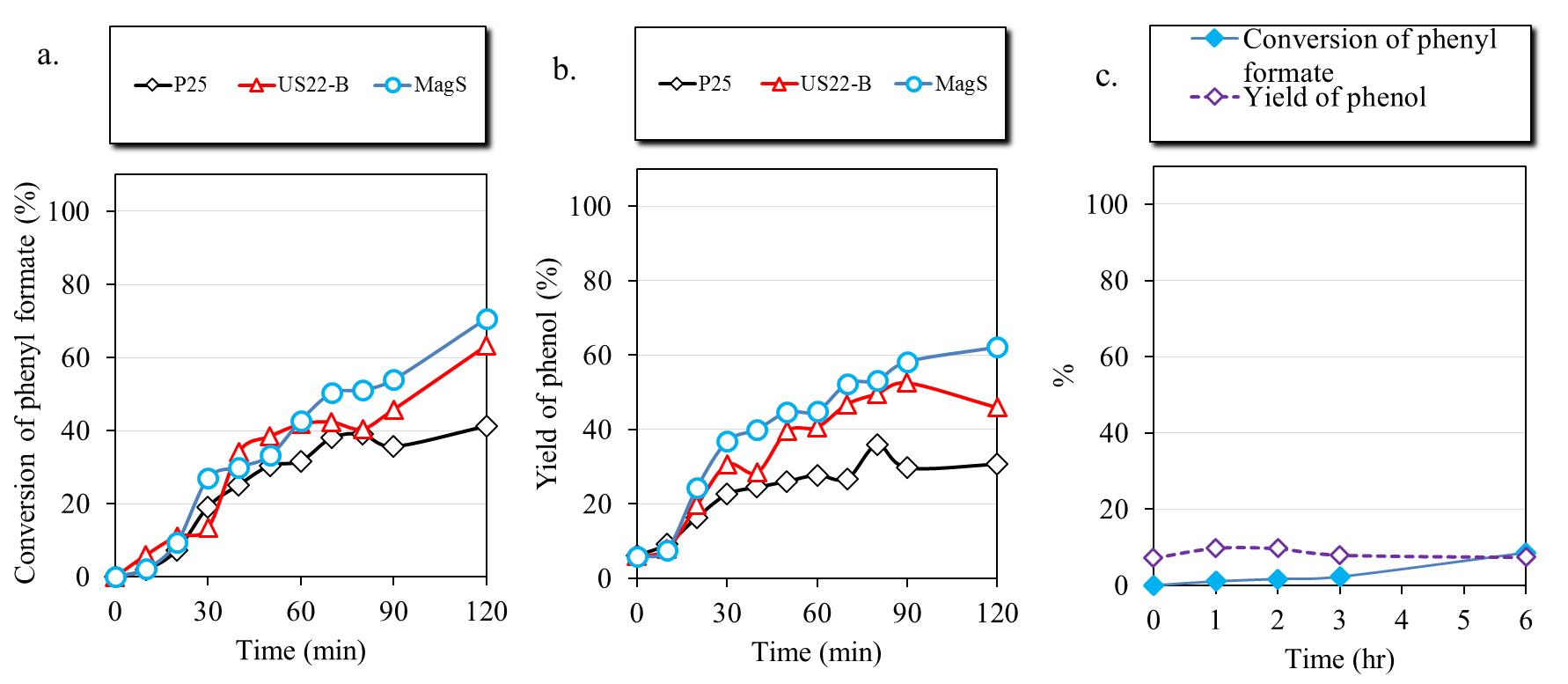


**Figure S14.** Adsorption/desorption experiments for Ph-OCHO, conversion of Ph-OCHO (a) and yield of phenol (b); photolysis of Ph-OCHO (c).


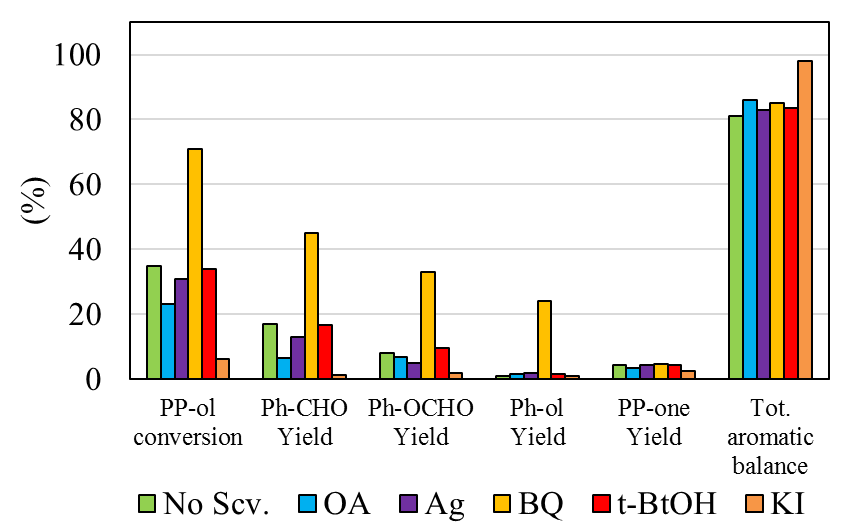


**Figure S15.** Photocatalytic study of MagS sample by the addition of various scavengers for the various species.
